# Cathelicidin promotes inflammation by enabling binding of self-RNA to cell surface scavenger receptors

**DOI:** 10.1038/s41598-018-22409-3

**Published:** 2018-03-05

**Authors:** Toshiya Takahashi, Nikhil Nitin Kulkarni, Ernest Y Lee, Ling-juan Zhang, Gerard C. L. Wong, Richard L. Gallo

**Affiliations:** 1 Department of Dermatology, University of California, San Diego, La Jolla, CA 92037 United States; 2 Department of Bioengineering, University of California, Los Angeles, Los Angeles, CA 90095 United States

## Abstract

Under homeostatic conditions the release of self-RNA from dying cells does not promote inflammation. However, following injury or inflammatory skin diseases such as psoriasis and rosacea, expression of the cathelicidin antimicrobial peptide LL37 breaks tolerance to self-nucleic acids and triggers inflammation. Here we report that LL37 enables keratinocytes and macrophages to recognize self-non-coding U1 RNA by facilitating binding to cell surface scavenger receptors that enable recognition by nucleic acid pattern recognition receptors within the cell. The interaction of LL37 with scavenger receptors was confirmed in human psoriatic skin, and the ability of LL37 to stimulate expression of interleukin-6 and interferon-β1 was dependent on a 3-way binding interaction with scavenger receptors and subsequent clathrin-mediated endocytosis. These results demonstrate that the inflammatory activity of LL37 is mediated by a cell-surface-dependent interaction and provides important new insight into mechanisms that drive auto-inflammatory responses in the skin.

## Introduction

Antimicrobial peptides (AMPs) play an essential role in the immune defense of all organisms. In mammals, the cathelicidin family of AMPs is abundantly produced in or recruited to damaged tissues where they participate in immunity through multiple mechanisms that include direct killing of target microbes and activation of host cell defense responses^1,2^. Transcriptional and post-transcriptional processing regulates expression of human cathelicidin peptides, such as the active form LL37 released from neutrophils^3^. The nascent cathelicidin protein is inactive, and proteolytic processing by serine proteases forms multiple cathelicidin peptides including LL37^4^. The importance of expression and processing of LL37 has been highlighted due to the association of AMP expression with multiple human diseases including inflammatory bowel disease^5^, lung cancer^6^, asthma, cystic fibrosis, chronic obstructive pulmonary disease^7^, Alzheimer’s disease^8^, systemic sclerosis^9^, systemic lupus erythematosus, rheumatoid arthritis, atherosclerosis^10^, rosacea, psoriasis, and atopic dermatitis^11^. In many of these disorders, the presence of excess LL37 is thought to enhance the local tissue inflammatory response.

Several mechanisms have been proposed for how LL37 and other AMPs can trigger inflammation. These include the ability of LL37 to directly activate cell surface receptors, or to act as an autoantigen^12,13^. Of particular interest have been multiple observations that LL37 greatly enhances cell responses to self-nucleic acids released from damaged and dying cells. In this scenario DNA or RNA serves as a damage associated molecular pattern (DAMP), and the cathelicidin peptide breaks immune tolerance to the presence of the nucleic acid, permitting recognition by intracellular recognition systems within the endosome and cytosol such as Toll-like receptor (TLR) 3, 7, 8, 9, mitochondrial antiviral-signaling protein (MAVS) and stimulator of interferon genes (STING)^14–16^. Both direct and indirect evidence supports the critical role that LL37 plays in driving tissue inflammation including observations that the cellular expression pattern of LL37 in psoriasis directly overlaps with the expression of type-1 interferon^16^. It is unclear how LL37 enables recognition of nucleic acids, but the membrane activity of the peptide that enables its antimicrobial activity is thought to control its capacity to permit trans-membrane penetration of stimuli to activate the cellular response^17^.

In the present study, we investigated the mechanism by which cathelicidin induces cytokine expression. A peptide library derived from LL37 was systematically evaluated for the capacity to enable an immune response to U1 RNA, a human non-coding RNA that is released after skin injury^18^. We observed that the ability of a cathelicidin peptide to disrupt membranes is not a necessary condition for breaking immune tolerance. LL37 was shown to enable recognition of nucleic acids by a previously unknown binding process to facilitate interaction with cell surface scavenger receptors (SRs) and drive clathrin-dependent endocytosis. These findings uncover a critical step in the host response to tissue damage and provide a therapeutic opportunity to block undesirable auto-inflammatory reactions.

## Results

### The immune response to LL37 is not dependent on antimicrobial activity

**Figure 1:**
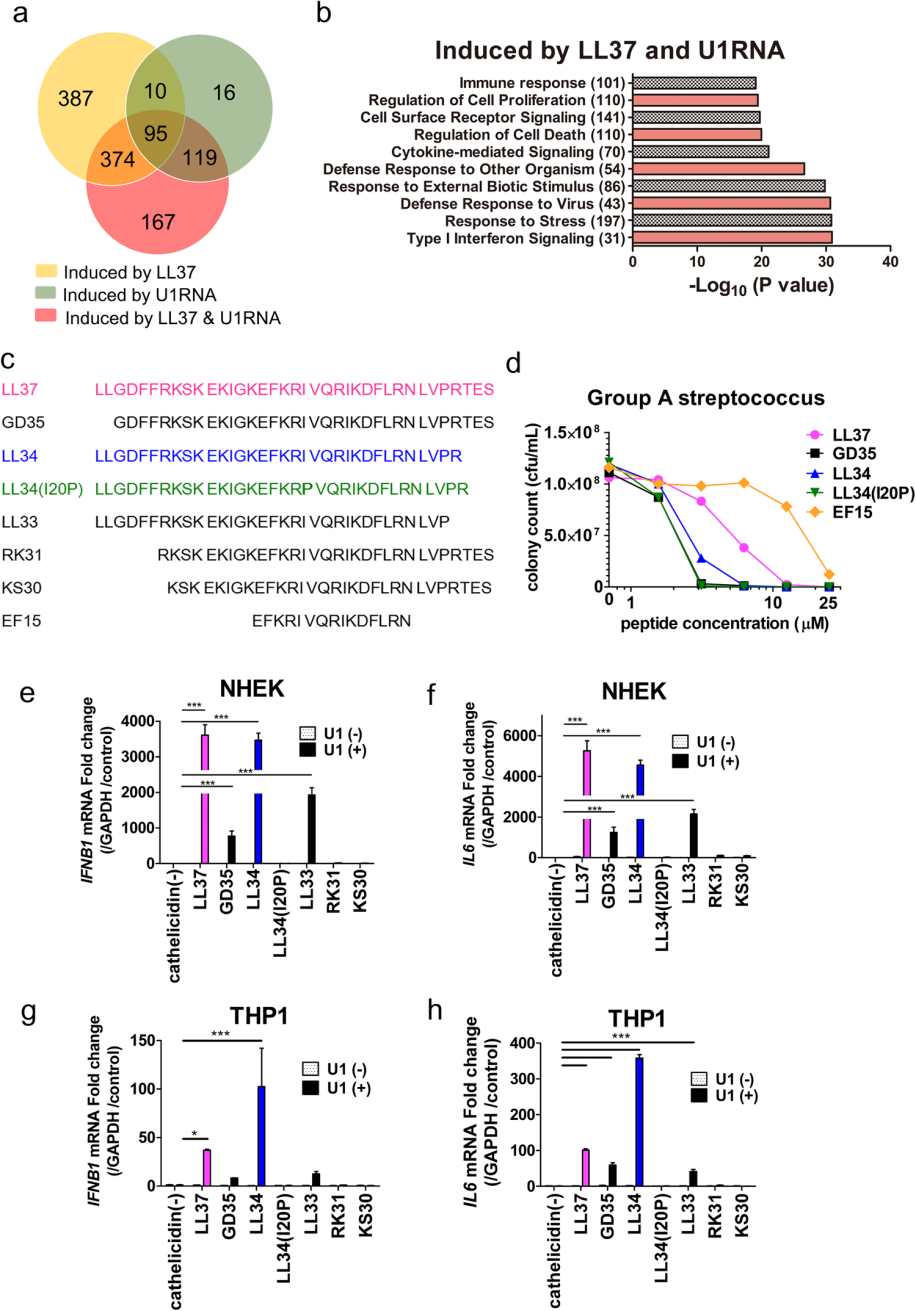
Inflammatory activity of cathelicidin can be dissociated from antimicrobial function (**a**) Transcriptomic analysis by RNASeq of primary cultures of normal human epidermal keratinocytes (NHEKs) treated with LL37 (2.5 μM) with or without U1 RNA (2.5 μg/mL) for 24 hours. Shown are gene sets induced > 2 fold over vehicle, and after treatment with LL37, U1 RNA, or the combination of LL37 + U1 RNA. (**b**) Gene Ontology analysis of gene sets from (**a**). The number of genes related to each biological process is indicated in parentheses. (**c**) Amino acid sequence of synthetic cathelicidin peptides used in this study. (**d**) Antimicrobial assay of cathelicidin peptides against group A streptococcus (GAS). GAS were incubated with various concentrations of each peptide (0–25 μM) for 6 hours, then surviving colonies counted after plating on Todd Hewitt Broth gel. (**e**) IFN-β1 mRNA abundance and (**f**) IL-6 mRNA abundance in NHEKs after treatment with cathelicidin peptides (2.5 μM) for 10 minutes, then stimulated with U1 RNA (2.5 μg/mL) for a further 6 hours. (n = 3). (**g**) IFN-β1 mRNA abundance and (**h**) IL-6 mRNA abundance in polymethylacrylate (PMA)-treated THP1 cells after treatment with cathelicidin peptides (3 μM) for 10 minutes, then stimulated with U1 RNA (12.5 μg/mL) overnight. (n = 3). Data presented are from one representative experiment of at least two independent experiments. Error bars are SEM of three biological replicates. *P < 0.05, **P < 0.01, ***P < 0.001 by two-way ANOVA with Bonferroni’s post-hoc test. See also Fig. S1.

The human cathelicidin antimicrobial peptide LL37 is an amphipathic cationic peptide that has dual host defense functions; it kills bacteria and promotes inflammation^19^. The function of LL37 to stimulate inflammation has been thought to be tied to its membrane activity where it can activate G-coupled receptors such as formyl peptide receptor 2 (FPR2, FPRL1)^12^, and enable cytosolic entry of extracellular nucleic acids^20^. To better understand the mechanism by which LL37 enables inflammatory responses, we performed RNA-sequencing to measure the transcriptome-wide effects of LL37 on primary human keratinocytes (NHEK) in the presence and absence of synthetic U1 RNA, an abundant non-coding RNA (ncRNA) that is released upon tissue damage^18,21^. One hundred and sixty seven genes were uniquely increased by 2-fold or more after exposure to the combination of LL37 and U1 RNA (Fig. 1a), and gene ontology analysis established that a combination of LL37 and U1 RNA was a significant stimulus of an epidermal inflammatory and defense response with a notable Type 1 interferon signature (Fig. 1b).

The structure of LL37 consists of a α-helix motif spanning residues 2–31 followed by a disordered C-terminal tail^22^. Having defined the transcriptional response of NHEK to ncRNA in the presence of LL37, we next synthesized a small series of peptides designed to interrogate the function of the N- and C-terminal domains, as well as determine the potential role of the α-helix by substitution of a proline to destabilize the peptide at position 20 (Fig. 1c). The positive charge and amphipathic structure of most cathelicidin peptides enable them to kill microbes by interacting with negatively charged phospholipid head groups and hydrophobic fatty acids in microbial membranes^23–25^, as well as influence the fluidity of dendritic cell and keratinocyte membranes^26–28^. Analysis of antimicrobial activity of these peptides against group A *Streptococcus* (GAS) showed that the synthetic peptides GD35, LL34 and LL34(I20P) had increased antimicrobial activity compared to LL37, whereas shorter fragments such as EF15 lost potency (Fig. 1d). As expected, antimicrobial activity correlated with the capacity of the peptides to permeabilize cell membranes as directly visualized by dye exclusion (Supplemental Fig. 1a). However, measurement of the cytokine response of NHEKs or THP1 cells (a human monocyte/macrophage cell line), revealed that the membrane activity of a peptide does not correlate with the capacity to enable U1 RNA to amplify interferon β1 (IFN-β1) or interleukin-6 (IL-6) expression (Fig. 1e–h and Supplementary Fig. 1b–g). Notably, despite large responses to U1 RNA enabled by the presence of LL37 or LL34, the LL34(I20P) peptide was consistently inactive. These results show that immunological activity can be clearly dissociated from membrane activity, or at least that the two activities are not codependent with one another.

### Cytokine responsiveness correlates with cathelicidin-enabled dsRNA binding to cells

**Figure 2:**
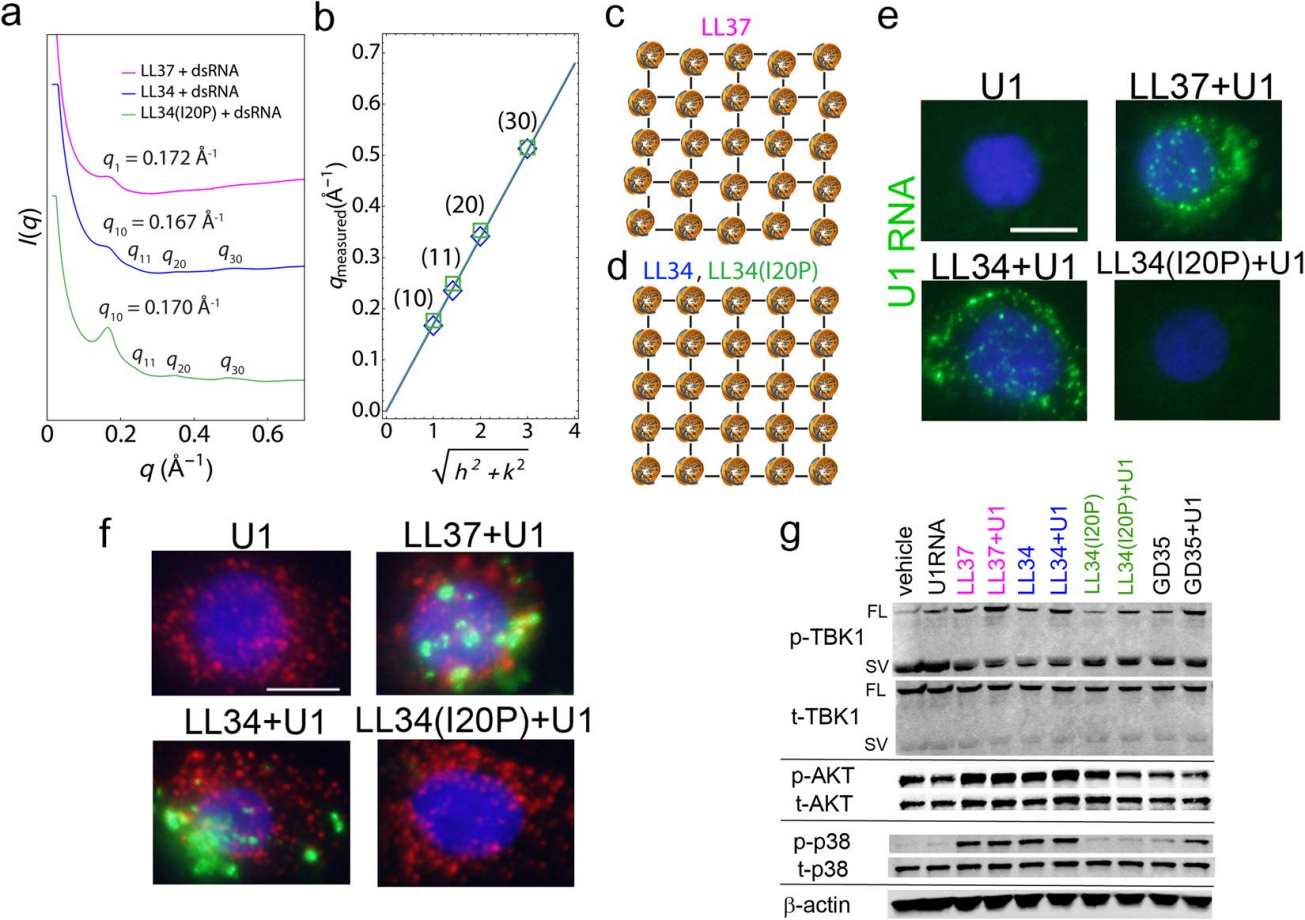
Structure and cell binding of cathelicidin and dsRNA. (**a**) Structures of LL37, LL34, and LL34(I20P) bound to dsRNA were solved using SAXS. SAXS data is plotted as the scattering intensity *I*(*q*) vs. the momentum transfer vector *q*. LL34 (blue) and LL34(I20P) (green) condense dsRNA into square lattices with first peak positions of *q*_10_ = 0.167 Å^−1^ and 0.170 Å^−1^, respectively. Higher order Bragg reflections due to scattering of the square lattice of LL34 and LL34(I20P) are labeled for both complexes. In comparison, LL37 (magenta) condenses dsRNA into a columnar lattice with short-ranged order, with a first peak position of *q*_1_ = 0.172 Å^−1^. (**b**) Linear fits of Bragg peak positions indicate that LL34-dsRNA (blue) and LL34(I20P)-dsRNA (green) complexes have inter-dsRNA spacings of 3.76 nm and 3.70 nm, respectively. Each reflection corresponding to the peaks in **a** for LL34 and LL34(I20P) are labeled. Close overlap of the two linear fits indicate that LL34-dsRNA and LL34(I20P)-dsRNA complexes have similar unit cell structures and inter-dsRNA spacings. End-on views along the dsRNA long-axis of the columnar (**c**) LL37-dsRNA and (**d**) LL34-dsRNA or LL34(I20P)-dsRNA complexes are shown. In (**d**), LL34-dsRNA and LL34(I20P)-dsRNA both form square columnar structures with similar unit cell architectures. LL37-dsRNA and d has an inter-dsRNA spacing of 3.65 nm, while LL34-dsRNA and LL34(I20P) have inter-dsRNA spacings of 3.76 nm and 3.70 nm. (**e**) NHEKs cultured at 4 °C for 1 hour with U1 RNA and cathelicidin peptides LL37, LL34 and LL34(I20P) each at (2.5 μM). Green staining is U1 RNA, blue is DAPI staining of nucleus. Scale bar represents 10 μm.(**f**) NHEKs cultured at 37 °C for 4 hours with U1 RNA and cathelicidin peptide. Early endosomal antigen 1 (EEA1) is stained red, U1 RNA is stained green and nuclei were visualized with DAPI (blue). Scale bar represents 10 μm. (**g**) NHEKs were pretreated with cathelicidin peptides (2.5 μM) for 10 minutes, then stimulated with U1 RNA (2.5 μg/mL) for a further 30 minutes (for AKT and p38) or 2 hours (for TBK and β−actin). Cell extracts were subjected to immunoblotting analyses using indicated antibodies after cutting each membrane at 50 kDa (30 minutes upper: AKT, 30 minutes below: p38, 2 hours upper: TBK1, and 2 hours below: β−actin). Full-length blots are presented in Supplementary Fig. 2d. Abbreviations: SV: spliced variant; FL: full length. Data presented are from one representative experiment of at least two independent experiments. See also Fig. S2.

To investigate whether the inability of LL34(I20P) to induce cytokine expression was due to a lack of binding to dsRNA, we conducted synchrotron small-angle X-ray scattering (SAXS) experiments to solve self-assembled structures of LL37-dsRNA, LL34-dsRNA, and LL34(I20P)-dsRNA complexes. Peptides were mixed with dsRNA at specific peptide-to-dsRNA stoichiometric rations, and the resulting peptide-dsRNA complexes were characterized with SAXS. Diffraction patterns show that LL37, LL34, and LL34(I20P) intercalate between dsRNA and crosslink them into ordered nanocrystalline bundles (Fig. 2a–d). Interestingly, recent work has shown that similarly structured LL37-dsDNA complexes can lead to significant amplification of TLR9 activation in plasmacytoid dendritic cells^29,30^. LL37 organized dsRNA into a columnar structure with short-ranged order at an average inter-dsRNA spacing of 3.65 nm (Fig. 2a,c). In comparison, LL34 and LL34(I20P) both organized dsRNA into nanocrystalline square columnar lattices with similar lattice parameters (Fig. 2a,b,d). This structure is consistent with close contact and charge compensation between the cationic peptides and anionic dsRNA, with a spatial periodicity of 3.76 nm and 3.70 nm between adjacent dsRNA within the complex (respective nanocrystal sizes of *L* = 9.0 nm and 16.6 nm). Interestingly, the structure of the LL34(I20P)-dsRNA complex is consistent with the capacity to enable dsRNA to be recognized by TLR3^31^. Therefore, we concluded that it was unlikely that the loss of immunological activity of LL34(I20P) was due to disruption of binding and structured complex formation with dsRNA or interference with TLR3 recognition.

We next hypothesized that the failure of LL34(I20P)-dsRNA to induce cytokines could be due to an inability of the complex to bind to the cell surface. To test this, NHEKs were exposed to U1 RNA at 4 °C for 1 hour before washing. LL37 and LL34 each showed the capacity to enable U1 RNA to bind to the cell, but LL34(I20P) did not (Fig. 2e**)**. Similarly, when NHEKs or THP1 cells were cultured with U1 RNA at 37 °C for 4 hours, LL37 and LL34 induced partial co-association with the early endosome while LL34(I20P) did not (Fig. 2f, Supplemental Fig. 2a). Partial co-localization of U1 RNA and early endosome suggests the possibility that some U1 RNA was also present on the cell surface. Partial cell surface localization is supported by analysis of intact cells as shown in Fig. 2e. Consistent with entry of dsRNA into the cell, phosphorylation in the TANK-Binding Kinase 1 (TBK1) - AKT serine/threonine kinase 1 (AKT) - interferon regulatory factor 3 (IRF3) pathway, and p38 mitogen-activated protein kinase (MAPK), was increased in response to LL37 and LL34 but not LL34(I20P) (Fig. 2g, Supplementary Fig. 2d). Similar to a previous report^16^, both LL37 and LL34 appeared to diminish phosphorylation of the small splice variant of TBK1 which negatively regulates IRF3 phosphorylation. FPR2 activity was also investigated due to its reported capacity to be activated by LL37^12^. However, treatment of NHEKs with pertussis toxin (an inhibitor of G-protein coupled receptors including FPR2), or WRW4 (selective antagonist for FPR2) did not decrease expression of IL-6 (Supplementary Fig. 2b,c). These results suggested that the ability of cathelicidin to enable dsRNA to associate with the cell and permit subsequent entry into the endosome was critical for activation of the cytokine response whereas activation of G-coupled receptors was not.

### LL37 enables dsRNA to bind to scavenger receptors

**Figure 3:**
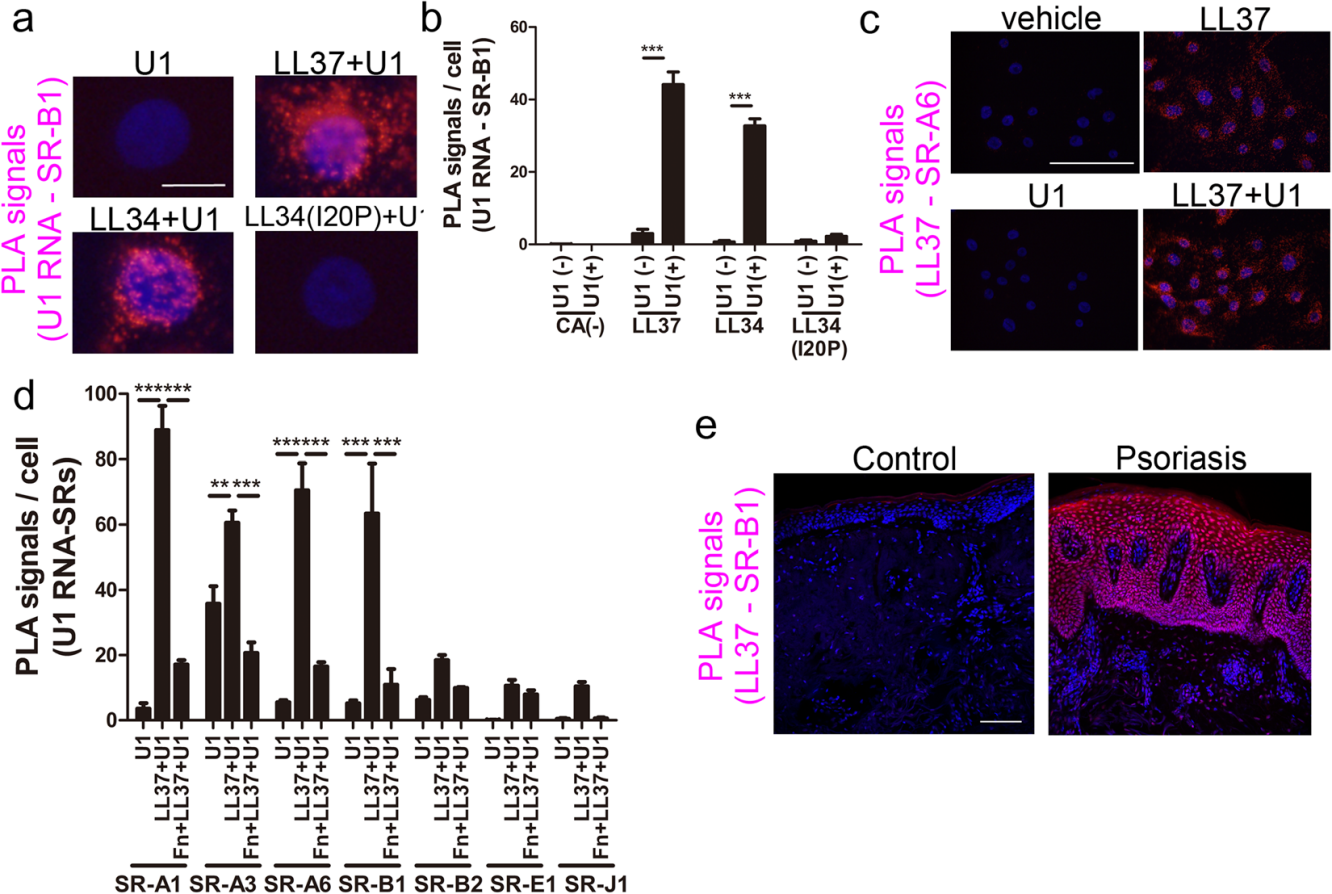
Cathelicidin enables dsRNA to associate with scavenger receptors. (**a**) Proximity ligation assay for SR-B1 and U1 RNA. NHEKs were cultured with various cathelicidin peptides (2.5 μM) for 10 minutes, then with biotinylated U1 RNA (2.5 μg/mL) at 4 °C for 1 hour before physical proximity of SR-B1 and U1 RNA was determined using a fluorescence-based PLA that produces a red fluorescent signal. Nuclei (blue) are counterstained with DAPI. Scale bar represents 10 μm. (**b**) Signal count of (**a**) in 5 visual fields. (**c**) Proximity ligation assay as in (**a**) but reaction is detecting proximity of LL37 and SR-A6. Scale bar represents 100 μm. (**d**) Signal count of proximity ligation assay performed as in (**a**), but between U1 RNA and the indicated scavenger receptors, each count is PLA complexes observed in 5 visual fields. (**e**) Skin sections from healthy subject (control), or lesional skin from a psoriasis patient (Psoriasis) were fixed and PLA performed for LL37 and SR-B1. Red fluorescent signal defines cells where LL37 and SR-B1 are within <40 nm of each other. Nuclei (blue) are counterstained with DAPI. Scale bars represent 100 μm. Data presented are from one representative experiment of at least two independent experiments. Error bars are SEM of three biological replicates. *P < 0.05, **P < 0.01, ***P < 0.001 by two-way ANOVA with Bonferroni’s post-hoc test. See also Fig. S3.

Scavenger receptors are abundant cell surface proteins that are known to associate with a variety of ligands including nucleic acids^32,33^ and can promote endocytosis^34^. Scavenger receptors SR-A6 and SR-B1 have been shown to be expressed on keratinocytes and to bind herpes simplex virus-1 to promote internalization^35,36^. Analysis of NHEKs by immunofluorescence suggested that the presence of LL37 or LL34 enabled U1 RNA to localize with SR-B1 whereas LL34(I20P) did not (Supplementary Fig. 3a). To stringently examine if cathelicidin peptides promote binding of dsRNA to scavenger receptors, proximity ligation assays (PLA) were performed to determine if binding occurs between U1 RNA and SR-B1 or SR-A6 (spatial correlation <40 nm). LL37 or LL34 enabled close association between U1 RNA and both scavenger receptors, but this binding did not take place in the absence of these peptides or in the presence of LL34(I20P) (Fig. 3a,b, Supplementary Fig. 3b,c). PLA was also performed between LL37 and SR-A6 (Fig. 3c), between other scavenger receptors and U1 RNA, and on THP1 cells (Supplementary Fig. 3d,e). U1 RNA bound to multiple scavenger receptors and in all cases this binding was dependent on presence of LL37 (Fig. 3d, and Supplementary Fig. 3d,e). Dynamic clustering of SR-B1 was observed on the surface of NHEK after exposure to LL37 and U1 RNA (Supplementary Fig. 3f and Supplementary Video a,b). Fucoidan, a competitive inhibitor of the scavenger-receptor family^37^, also significantly diminished binding (Fig. 3d), although fucoidan also showed preferential binding of LL37 to fucoidan over binding to dsRNA, forming structures with a clearly different diffraction signature in SAXS experiment (data not shown).

The close association of scavenger receptors with LL37 was also demonstrated in human skin affected with psoriasis. Histological assessment and PLA revealed that LL37 was closely associated with SR-B1 in this disease (Fig. 3e, Supplementary Fig. 3g). Previously, the expression of LL37 on keratinocytes of whole skin involved with psoriasis was found to exactly match with the keratinocytes that expressed IFN-β1^16^. Taken together, this suggests that IFN-β1 expression occurs only in cells where LL37 is able to bind with with SR-B1.

### Scavenger receptors are necessary for the immune function of LL37

**Figure 4:**
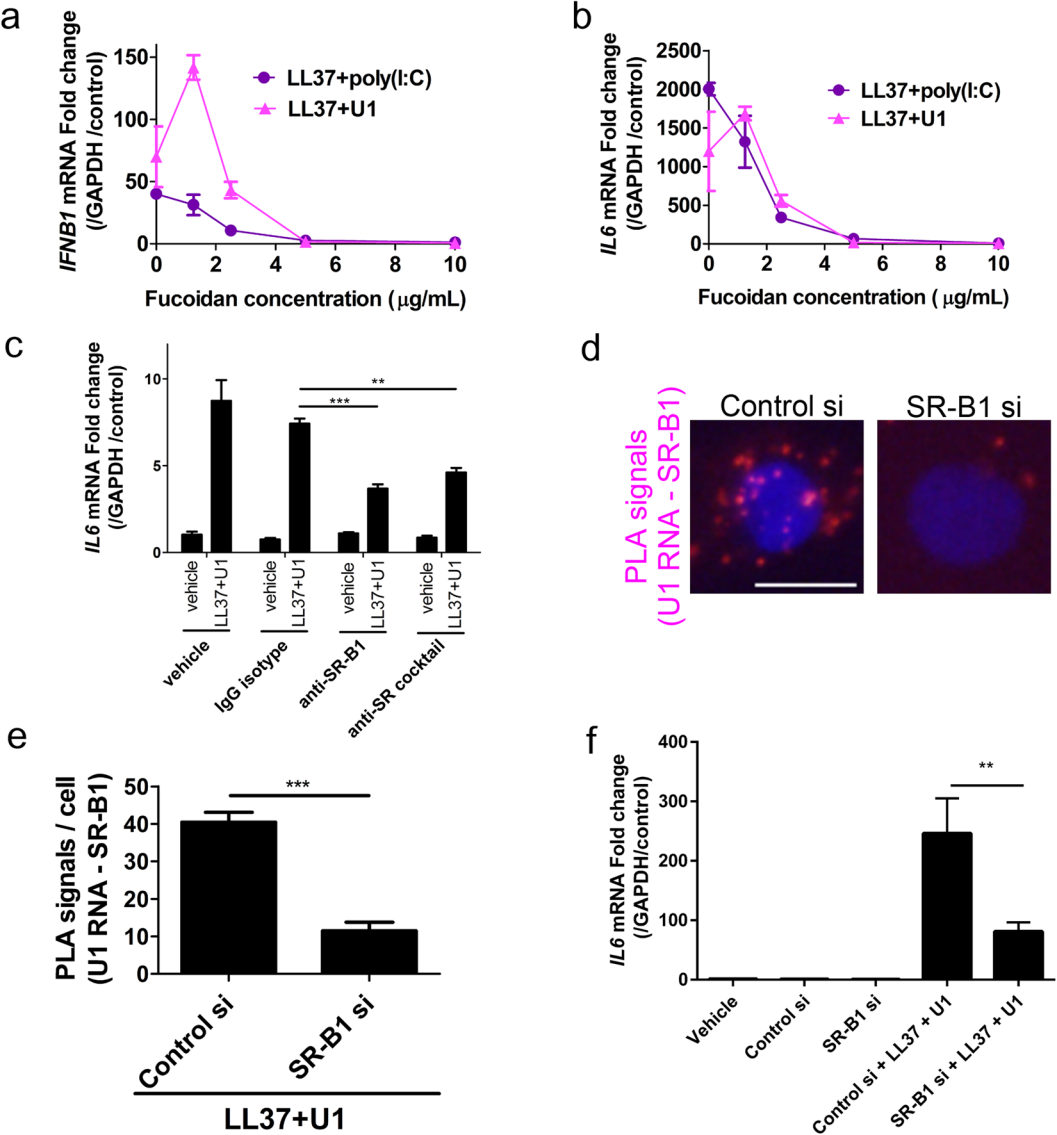
Scavenger receptors are necessary for immune response to LL37 (**a**) IFN-β1 mRNA measured in NHEKs in the presence of various concentrations of Fucoidan and addition of LL37 and U1 RNA (triangles) or LL37 and poly(I:C) (circles). (**b**) IL-6 mRNA measured in NHEK in the presence of various concentrations of fucoidan and addition of LL37 and U1 RNA (triangles) or LL37 and poly(I:C) (circles). (**c**) IL-6 mRNA in NHEKs cultured in vehicle alone or with antibodies for SR-B1, a mixture of antibodies for 7 scavenger receptors, SR-A1, A3, A6, B1, B2, E1, and J1 (SR cocktail), or IgG isotype (control) at 12 μg/ml for 5 minutes following cycloheximide (10 μg/mL) for 30 minutes, then treated with LL37 (2.5 μM) for 10 minutes, and U1 RNA (2.5 μg/mL) for a further 6 hours. (**d**) Proximity ligation assay of U1 RNA and SR-B1 in NHEKs treated with control siRNA or siRNA targeting SR-B1. Scale bar represents 10 μm. (**e**) Signal count of (**d**) in 5 visual fields. (**f**) IL-6 mRNA in NHEKs following siRNA knockdown of gene coding SR-B1 (SCARB1). Cells were treated with LL37 (2.5 μM) and U1 RNA (2.5 μg/mL) for a further 6 hours. (n = 3). Data presented are from one representative experiment of at least two independent experiments. Error bars are SEM of three biological replicates. *P < 0.05, **P < 0.01, ***P *<* 0.001 by two-way ANOVA with Bonferroni’s post-hoc test. See also Fig. S4.

To determine the functional significance of the cathelicidin-dependent binding of dsRNA to scavenger receptors, we tested the effect of fucoidan on inflammatory cytokine expression stimulated with LL37 and dsRNA. Fucoidan is a nonspecific inhibitor of multiple scavenger receptors and potently inhibited the capacity of LL37 to induce cytokines when combined with either U1 RNA or synthetic viral dsRNA poly(I:C) (Fig. 4a,b, Supplemental Fig. 4a–g).

Since the inhibitory action of Fucoidan could also be due to disruption of the association of U1RNA with LL37, additional experiments to specifically target scavenger receptors were performed. The expression of inflammatory cytokines by LL37 and U1RNA was inhibited by neutralizing antibody against SR-B1, or a mixture of seven anti-scavenger receptors antibodies (SR-A1, A3, A6, B1, B2, E1, and J1), compared to IgG isotype controls (Fig. 4c and Supplementary Fig. 4h). siRNA mediated knock down was also performed against scavenger receptors to further investigate the dependence on scavenger receptors for LL37 function (Fig. 4d–f, Supplementary Fig. 4i). After silencing SR-B1 and addition of LL37, PLA showed reduced appearance of the U1 RNA-SR-B1 complex (Fig. 4d,e). Cytokine expression was also significantly suppressed following SR-B1 knockdown (Fig. 4f). Silencing of SR-A6 did not show a significant decrease in PLA assay or cytokine response (data not shown). Taken together, we conclude that cathelicidin-dependent dsRNA activation of inflammatory cytokine expression is dependent on scavenger receptors with SR-B1 likely playing a major role.

### Immune function of LL37 on U1 RNA is dependent on clathrin-mediated endocytosis

**Figure 5:**
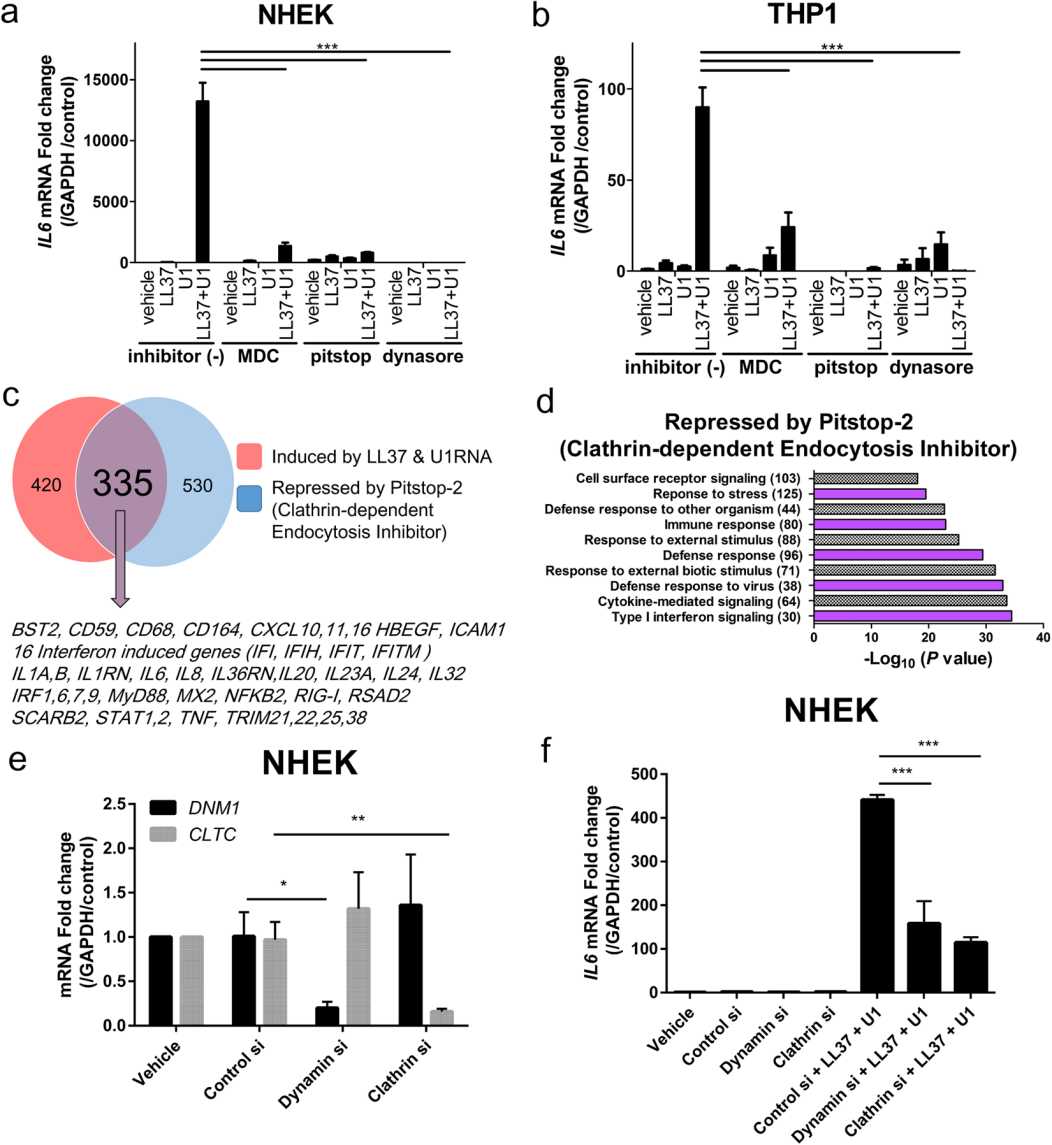
Immune response to LL37 requires clathrin-dependent endocytosis (**a**) IL-6 mRNA in NHEKs cultured with various endocytosis inhibitors (Monodansylcadaverine (MDC): 200 μM, Pitstop-2^TM^ 25 μM, dynasore: 80 μM) for 30 minutes, treated with LL37 (2.5 μM) and U1 RNA (2.5 μg/mL) for a further 6 hours. (n = 3). (**b**) IL-6 mRNA in PMA-treated THP1 after treatment with cathelicidin peptides (3 μM) for 10 minutes, then stimulated with U1 RNA (12.5 μg/mL) overnight. (n = 3). (**c**) Gene sets induced in NHEK by LL37 + U1 RNA (fold change >2 versus vehicle control) and genes of identically treated cells first repressed by Pitstop-2^TM^ (25 μM) (fold change < −1.5). (**d**) Gene Ontology analysis gene set in **c** that was both induced by LL37 + U1 RNA and also inhibited by Pitstop-2^TM^. The number of genes related to each biological process is indicated in parentheses. (**e**) Dynamin (DNM1) mRNA and clathrin (CLTC) mRNA measured in NHEKs after siRNA targeting each gene. (n = 3). (**f**) IL-6 mRNA in NHEKs following siRNA targeting of DNM1 or CLTC, and addition of LL37 (2.5 μM) and U1 RNA (2.5 μg/mL) for 6 hours. (n = 3). Data presented are from one representative experiment of at least two independent experiments. Error bars are SEM of three biological replicates. *P < 0.05, **P < 0.01, ***P *<* 0.001 by two-way ANOVA with Bonferroni’s post-hoc test. See also Fig. S5.

Classical ligands for scavenger receptors enter endosomes by endocytosis^33^. Since LL37 mediated dsRNA association with early endosomal antigen 1 (EEA1) (Fig. 2f) and permitted close association with scavenger receptors (Figs 3 and 4), we next tested if the cytokine inducing activity enabled by LL37 was dependent on clathrin-mediated endocytosis. Both NHEKs and THP1 treated with endocytosis inhibitors Monodansylcadaverine (MDC), Pitstop-2^TM^, or dynasore^38,39^ showed a significant inhibition of the expression of inflammatory cytokines (Fig. 5a,b and Supplementary Fig. 5a–c). Full transcriptome analysis following addition of a selective clathrin inhibitor (pitstop-2) confirmed endocytosis was required for the expression genes induced by LL37 and U1 RNA that enable dsRNA to initiate an inflammatory response (Fig. 5c,d). Phosphorylation of TBK1-AKT-IRF3 and p38 MAPK was also inhibited by endocytosis inhibitors (Supplementary Fig. 5d). siRNA knockdown of dynamin (DNM1) or clathrin (CLTC) also reduced expression of cytokines by U1 RNA and LL37 (Fig. 5e,f) and validated the role of these critical genes in endocytosis. Macropinocytosis inhibitor IPA-3 did not inhibit IFN-β1 and IL-6 expression (Supplementary Fig. 5e,f).

## Discussion

Cathelicidin is post-translationally processed into several peptide forms, but the 37-amino acid peptide (LL37) is the form most abundantly produced by the skin in response to injury or infection^40^. LL37 can not only directly kill some microbes, but can also potently amplify the inflammatory response^2^. We sought to understand how an inflammatory cytokine response is triggered by LL37 because it is both necessary for appropriate immune defense and harmful when promoting auto-inflammation in human diseases^7–10,41,42^. A structure-function analysis revealed that the membrane disruptive activity of LL37 could be dissociated from its capacity to enable recognition of dsRNA. Subsequent comparison of the properties of active and inactive peptides further showed that LL37 promotes cytokine expression in human cells by facilitating dsRNA binding to cell surface scavenger receptors rather than directly enhancing membrane permeability. The complex of LL37, scavenger receptor and nucleic acid then enables endocytosis to trigger the canonical signaling pathway characteristic of inflammatory activation by cathelicidin.

Interestingly, although LL34-dsRNA and LL34(I20P)-dsRNA complexes show similar inter-dsRNA spacings, they exhibit quite different behavior with respect to binding to surface scavenger receptors and subsequent induction of endocytosis. The LL34(I20P)-dsRNA complexes are significantly better ordered with more intense diffraction peaks and stronger crystalline ordering compared to either LL34-dsRNA complexes or LL37-dsRNA complexes, both of which bind to SRs and induce endocytosis. One possibility is that the I20P mutation induces more ordered crystalline packing in the complex and more “solid-like” elastic behavior in the complex, therefore resulting in less-deformable binding surfaces that do not accommodate dual binding to both SRs and TLR3. Recent results have shown that amyloid-DNA complexes endocytose *via* TLR2 and subsequently bind to endosomal TLR9 in macrophages^43^. Another possibility is that that the I20P mutation has disrupted a binding motif in the nanocrystalline complex that is necessary for SR binding. What seems to be clear is that LL37 performs a sophisticated process of “innate immune vetting”, whereby the presence of LL37 in the local environment determines if host nucleic acids become pro-inflammatory. More generally, assembly of immune ligands into ordered complexes with different structures enables differential binding to various receptors, thereby mediating distinct immune outcomes. This expression of cathelicidin is therefore a critical event that initiates appropriate inflammation during acute infection or injury, but drives inappropriate inflammation in auto-inflammatory diseases.

Our analysis of the global transcriptional response of keratinocytes indicated that the combination of LL37 and U1 RNA induced a characteristic gene signature that could be clearly distinguished from responses to peptide or U1 RNA alone. Identification of this gene set permitted selection of IL-6 and IFN-β as representative cytokines to screen a peptide library for activity. A cathelicidin peptide derived from the N-terminal 34 amino acids of LL37 (LL34) had potent antimicrobial activity similar to LL37 with strong antimicrobial activity against GAS (minimal bacteriocidal concentration (MBC) ca. 5 uM), and strong capacity to induce IL-6 and IFN-β mRNA (ca. 3000-fold). Remarkably, substitution of isoleucine for proline at position 20 abolished cytokine induction activity but did not decrease the antimicrobial activity of the peptide (LL34(I20P)). Although the proline substitution is predicted to destabilize the α-helix structure of LL34, it did not affect the peptide’s ability to penetrate bacterial or mammalian membranes. Importantly however, the native isoleucine was required for dsRNA to trigger a cytokine response. This demonstrates that the sequence and structural rules for antimicrobial activity of AMPs are distinct from the rules encoding proinflammatory activity via dsRNA binding, consistent with prior functional observations of cathelicidin peptides.

Diverse antimicrobial peptides are encoded by exon 4 of cathelicidin in other species^44,45^, and diverse antimicrobial peptides are generated in humans by posttranscriptional processing via serine proteases^4^. In contrast, only LL37 has shown clear capacity to potentiate host inflammation^41,46^.

The observation that a point mutation in cathelicidin abolished dsRNA entry but conserved membrane activity led us to investigate alternative hypotheses for how LL37 promotes cytokine response. dsRNA at high concentrations or of viral origin are known to directly enter cells through scavenger receptors and subsequently induce inflammation through cytosolic or endosomal recognition receptors such as RIG-1-like receptors or TLR3^47–49^. For example, the synthetic viral dsRNA poly(I:C) can trigger inflammatory responses alone^16,50^. Our observations with poly(I:C) were consistent with this but clearly showed that without an appropriately structured cathelicidin peptide, synthetic U1 RNA had little activity. Multiple experimental approaches showed that U1 RNA will only bind and activate cells when cathelicidin enables interaction with scavenger receptors. These techniques included co-localization, proximity ligation assay, fucoidan or antibody mediated blockade of scavenger receptors, and siRNA-targeted knockdown of scavenger receptor mRNA. Further support for the necessity for LL37 to interact with scavenger receptors came with demonstration of the dependence of cytokine production on clathrin-mediated endocytosis, a hallmark of scavenger receptor function^51,52^. Indeed, cathelicidin has been reported to enhance poly(I:C) activity through FPR2 and depended on clathrin-independent endocytosis^53^. Our results did not suggest that activation of FPR2 was needed for activity or that the cathelicidin-dsRNA complex was only limited to one class of scavenger receptors. The association of multiple scavenger receptors with LL37 observed by PLA is consistent with observations that scavenger receptors can share functional properties despite little or no homology among classes^33^. Future investigations are required to further measure the affinity for the different cell surface scavenger receptors, define specific structural domains required for each binding element of the complex, and determine if other components of the cell membrane are needed for this process.

These findings have important implications for better understanding normal host defense and human diseases that are exacerbated by the presence of LL37. Using proximity ligation assay we directly demonstrated that LL37 associates with scavenger receptors in human skin. Scavenger receptors are considered to be a subclass of the membrane-bound pattern recognition receptors^33^. The pattern of colocalization of LL37 and SR-B1 as shown in Fig. 3e corresponded with the pattern of type 1 interferon previously shown to be induced in the epidermis of patients with psoriasis^16^. This pattern supports the hypothesis that the excessive inflammation that occurs in psoriasis is due to release of nucleic acids and amplification by excess LL37 in the tissue that promotes initial binding to scavenger receptors. We show this event can take place in response to dsRNA in keratinocytes and a monocyte/macrophage cell line, but LL37 may promote enhanced binding to scavenger receptors on other cell types and with ligands such as DNA, endogenous proteins and lipoproteins or molecules derived from microbes.

In summary, our findings provide new insight into how cathelicidin orchestrates the response to DAMPs to induce the production of inflammatory cytokines by both epithelial cells and macrophages. These results describe a mechanism for how excess cathelicidin can contribute to unwanted auto-inflammation. To our knowledge, a role for scavenger receptors in this process was not previously known. Importantly, with appreciation of the dependence on this interaction it becomes possible to devise new therapeutic interventions to block inappropriate inflammation driven by LL37.

## Methods

### Chemicals

Synthetic cathelicidin peptides were synthesized and purchased from Genemed synthesis Inc (San Antonio, TX). U1 RNA and biotinylated U1 RNA were generated by *in vitro* transcription as described previously^54^. Poly(I:C) and biotinylated poly(I:C) were purchased from Invivogen (San Diego, CA). MDC, dynasore, fucoidan, cycloheximide, Pertussis toxin from Bordetella pertussis, and saponin from quillaja bark were all purchased from Sigma-Aldrich (St Louis, MO). Pitstop 2^TM^ was purchased from Abcam (Cambridge, MA). Boc-MLF and Tocris WRW4 were purchased from Thermofisher Scientific (Waltham, MA).

### Antibodies

Antibodies in this study are shown on Table S1.

### Cell culture

Primary neonatal human epidermal keratinocytes (NHEKs) was purchased from ThermoFisher Scientific. NHEKs were grown in serum free EpiLife medium supplemented with 0.06 mM CaCl_2_, EpiLife Defined growth supplements (EDGS) (ThermoFisher Scientific) and antibiotics, and passage 3–5 cells were used for experiment. Cells at 60–80% confluence were starved overnight without EDGS prior to treatment. THP1 was purchased from American Type Culture Collection (ATCC) (Manassas, VA). THP1 was cultured in RPMI-1640 (Sigma) supplemented with 10% Hyclone fetal calf serum (Thermofisher Scientific), and antibiotics, and passage 3–5 cells were used for experiment. Cells at 60–80% confluence were differentiated by Phorbol 12-myristate 13-acetate (PMA, Sigma) for 24 hours and then starved overnight without Hyclone fetal calf serum prior to treatment.

### Synthesis of U1 RNA and biotinylated U1 RNA

First PCR reaction was performed to make DNA templates for U1 RNA synthesis, using AccuPower® TLA PCR PreMix (Bioneer, Daejeon, Korea) according to the manufacturer’s instructions, human genomic DNA (Promega, Madison, WI) as template, and primers (Forward T7: TAATACGACTCACTATAGGGATACTTA, Reverse T7: CAGGGGAAAGCGCGA). Following agarose gel running and purification of DNA templates using MinElute Gel Extraction Kits (Qiagen, Hilden, Germany) according to the manufacturer’s instructions, U1 RNA was synthesized, purified, and precipitated using AmpliScribe™ T7 High Yield Transcription Kit (Lucigen, Middleton, WI) according to the manufacturer’s instructions. Biotinylated U1 RNA was produced similarly, except that 1:2 mixture of biotin-16-UTP and UTP was used for synthesis of RNA.

### Quantitative real-time PCR

RNA was isolated from NHEKs using Purelink RNA isolation columns (ThermoFisher Scientific) according to the manufacturer’s instructions. RNA was quantified using a Nanodrop spectrophotometer (ThermoFisher Scientific), and up to 500 ng of RNA was reverse-transcribed using the iScript cDNA synthesis kit (Bio-Rad, Irvine, CA). Quantitative real-time PCR reactions were run on a CFX96 real-time detection system (Bio-Rad) using gene-specific primers and TaqMan probes (ThermoFisher Scientific). PCR primers and probes are shown on Table S2.

### Measurement of protein secretion

Cell culture supernatants were isolated, and cellular debris was removed by centrifugation at 600 *g* for 5 minutes. Cytokine protein concentrations were determined using BD OptEIA ELISA kits (BD Biosciences, San Diego, CA) or Milliplex MAP Immunoassay kits (EMD Millipore, Billerica, MA) according to the manufacturer’s instructions. Milliplex assays were analyzed on a MAGPIX instrument (Luminex Corporation, Austin, TX).

### Immunocytofluorescence

NHEKs were grown on 8-well chamber slides (Thermofisher Scientific). After indicated treatments, cells were fixed in 4% paraformaldehyde (PFA) (Thermofisher Scientific) for 10 minutes and blocked with 3% bovine serum albumin, 0.2 M Glycine (Sigma) with or without 1 mg/mL saponin (Sigma) prior to incubating with primary antibodies followed by appropriate 488- or 568-coupled secondary antibodies, or 488-streptavidin conjugate. Nuclei were counterstained with DAPI. All images were taken with an Olympus BX41 microscope (widefield), Zeiss LSM510 confocal microscope, or Nikon A1R Confocal STORM microscope as indicated.

### Protein extraction and immunoblotting analyses

NHEKs were lysed in a denaturing lysis buffer containing 20 mM HEPES pH 7.4, 250 mM NaCl, 2 mM EDTA, and 1% SDS supplemented with completed proteinase inhibitor cocktail as well as 50 mM sodium floride, 5mM N-ethylmaleimide, 100 μM hemin chloride to maximally preserve protein post-translational modifications as described previously^55^. Lysates were boiled for 3 minutes homogenized by sonication using digital sonifier (Branson Ultrasonics, Danbury, CT) followed by centrifugation to remove DNA and cell debris. Protein concentrations were measured by BCA protein assay kit (Thermofisher). For immunoblotting, 10–20 μg of protein was separated on a 10% Tris-Glycine precast gel (Biorad), transferred to PVDF membrane (Biorad), followed by immunoblotting using indicated primary antibodies followed by fluorescent secondary antibodies (LICOR Biosciences, Lincoln, NE) and imaging using fluorescent Odyssey System (LICOR Biosciences).

### Proximity ligation assay (PLA)

NHEKs seeded into 8 chamber slides (Thermo Fisher Scientific) were incubated with various combination of U1 RNA, poly(I:C), cathelicidin peptides, and fucoidan for 1 hour at 4 °C allowing binding, but not internalization. Unbound RNA was removed by washing with cold PBS. Cells were fixed with 4% PFA at 4 °C. Blocking buffer (Sigma) was used to prevent nonspecific antibody binding, and cells were incubated with two primary antibodies. Secondary antibodies conjugated with oligonucleotides were added, and hybridization, ligation, amplification and detection steps were performed according to the manufacturer’s instructions (Sigma) to generate an amplified fluorescent signal in areas where the antigens recognized by the two primary antibodies reside within less than 40 nm. Fluorescent PLA signals were evaluated using fluorescence microscopy (described above). Signals are counted by Duolink ImageTool software version 1.0.1.2 (Sigma-aldrich).

### RNA sequencing

Purified RNA was submitted to the University of California, San Diego (UCSD) Institute for Genomic Medicine core facility for library preparation and high-throughput next-generation sequencing. Libraries were constructed using TruSeq Stranded mRNA Library PrepKits (Illumina, San Diego, CA) and run on a HiSeq. 2500 instrument (Illumina). Raw data were analyzed using Partek Flow and Partek Genomics Suite software v6.0.17.0319 to determine transcript abundance and differentially expressed genes between samples. Gene Ontology analysis was performed using the Gene Ontology Consortium Database (http://geneontology.org/).

### *In vitro* antimicrobial assays

To determine MBC of cathelicidin peptides against Group A streptococcus (GAS), GAS (1 × 10^5^ CFU per mL) was incubated with cathelicidin peptides at various concentrations (1.5–25 μM) in PBS on a 96-well microplate (100 μL per well) for 6 hours under anaerobic conditions. The reaction mixture was diluted 1:10–1:10^6^ with PBS. MBC was determined by spotting the dilution (10 μL) on a Todd Hewitt broth agar plate for the counting of CFUs.

### siRNA-mediated gene knockdown

NHEK cells were transfected with siRNAs directed against SCARB1 (Dharmacon On-Target Plus, Life Technologies, Catalog No. SO-2605771G), Dynamin (Santacruz Biotechnologies, Catalog No. sc-43737) and clathrin (Santacruz Biotechnologies, Catalog No. sc-35067) at a final concentration of 10 nM according to manufacturer’s instructions. Briefly, the siRNA complexes were prepared in antibiotic free complete Epilife medium and incubated at room temperature for 5 minutes. Similarly, Lipofectamine RNAi max reagent (Life Technologies, Catalog No. 13778150) was diluted in antibiotic free complete Epilife medium at a concentration of 2.5% and incubated at room temperature for 5 minutes. The diluted siRNA and lipofectamine were combined, gently mixed and incubated for 20 minutes at room temperature to allow for formation of siRNA-Lipofectamine complexes. Following incubation, the siRNA-Lipotectamine complexes were added to the cells at a final siRNA concentration of 10 nM and containing Lipofectamine at a final concentration of 0.25%. After 24 hours of transfection medium was changed to complete Epilife medium with antibiotics and the cells were rested for a further 24 hours. After a total 48 hours of siRNA transfection, the cells were treated with LL37 (2.5 µM) and U1 RNA (1 µg/mL) for 6 hours, following which the cells were processed for gene expression analysis. SCARB1, Dynamin and Clathrin gene knockdown were assessed with quantitave real-time PCR after 48 hours of siRNA transfection.

### SAXS Experiments and Data Analysis

The structural phase diagrams of peptide-dsRNA complexes were mapped out by incubating peptides (10 mg/mL) with dsRNA (5 mg/mL) at specific charge ratios in microcentrifuge tubes as described above. After thorough mixing and centrifugation, precipitated complexes are hermetically sealed in 1.5 mm quartz capillaries (Hilgenberg GmbH, Mark-tubes). SAXS experiments were performed at the Stanford Synchrotron Radiation Lightsource (SSRL, Beamline 4–2) using monochromatic X-rays with an energy of 9 keV. A Rayonix MX225-HE detector (pixel size 73.2 μm) was used to measure the scattered radiation. Independent identical samples were prepared and measured over multiple separate experiments to ensure consistency. 2D powder diffraction patterns were integrated using the Nika 1.76^56^ package for Igor Pro 7.04 and FIT2D^57^. SAXS data were analyzed by plotting integrated scattering intensity against the momentum transfer *q* using Mathematica. Peak positions were measured by fitting diffraction peaks to a Lorentzian. Structures of complexes were solved by calculating ratios between the *q*-positions of all measured peaks and comparing them with the permitted reflections for known liquid-crystalline phases. The lattice parameter(s) of each phase were calculated by linear regression through points corresponding to measured and theoretical peaks. The lattice parameter *a* indicates the inter-dsRNA spacing between RNA columns. For each complex, the inter-dsRNA spacing *a* is estimated from the first peak position by the formula a = 2π/*q*_1_.

To determine the phase and lattice parameters for each nanocrystalline complex, we measure the q peak positions and relate them to the Miller indices for powdered averaged phases. ${q}_{hk}=\,\frac{2\pi }{a}\sqrt{{h}^{2}+{k}^{2}}$ for square columnar lattices and ${q}_{1}=\,\frac{2\pi }{a}$ for disordered columnar lattices. Square lattices were assigned based on fitting the measured q positions to the above equations. Typical square lattices will have reflections at *q*_10_ and *q*_11_ with a ratio of 1:√2. Procedures to assign these liquid-crystalline phases are similar to those found here^29,58^.

In addition to quantifying the lattice parameters and inter-dsRNA spacings in each complex, we also measured average domain size *L* of each complex. We approximated the structure factor peaks as squared-Lorentzian functions

1 $$S(q)=\frac{{w}^{3}}{4\pi {({|q-{q}_{0}|}^{2}+{(\frac{w}{2})}^{2})}^{2}};$$

where *q*_0_ is the location of the first peak, and *w* is the peak width^29^. The experimental SAXS data was background subtracted, and the first peak for each complex was fitted using nonlinear least-squares regression in Mathematica. The extracted value for peak width *w* can be related to the average linear domain size *L* using Warren’s approximation^59^. For the squared-Lorentzian lineshape, the domain size is related to *w* as follows: $L=\frac{{(8\pi )}^{\frac{1}{2}}}{\frac{w}{2}}$^60^.

### Statistical analysis

To compare means between more than two groups, a two-way ANOVA with Bonferroni’s post-hoc test was performed. All statistical analyses were performed using Prism GraphPad version 5.03 (Intuitive Software for Science, San Diego, CA). A value of *P* < 0.05 was considered significant, where *P < 0.05, **P < 0.01, ***P < 0.001. No statistical methods were used to predetermine sample size. The experiments were not randomized. The investigators were not blinded.

### Data availability

The data supporting the findings of the study are included in the Figures and Supplementary Information or can be obtained from the authors upon reasonable request.

## Electronic supplementary material

supplementary information

supplementary video a

supplementary video b

## References

[1] Zhang L-j, Gallo RL (2016). Antimicrobial peptides. Current Biology.

[2] Hancock REW, Haney EF, Gill EE (2016). The immunology of host defence peptides: beyond antimicrobial activity. Nature Reviews Immunology.

[3] Gudmundsson GH (1996). The human gene FALL39 and processing of the cathelin precursor to the antibacterial peptide LL‐37 in granulocytes. The FEBS Journal.

[4] Yamasaki K (2006). Kallikrein-mediated proteolysis regulates the antimicrobial effects of cathelicidins in skin. FASEB J.

[5] Geremia A, Biancheri P, Allan P, Corazza GR, Di Sabatino A (2014). Innate and adaptive immunity in inflammatory bowel disease. Autoimmunity Reviews.

[6] Li D (2014). Expression of the antimicrobial peptide cathelicidin in myeloid cells is required for lung tumor growth. Oncogene.

[7] Hiemstra PS, Amatngalim GD, van der Does AM, Taube C (2016). Antimicrobial peptides and innate lung defenses: role in infectious and noninfectious lung diseases and therapeutic applications. Chest.

[8] Welling MM, Nabuurs RJA, van der Weerd L (2015). Potential role of antimicrobial peptides in the early onset of Alzheimer’s disease. Alzheimer’s & Dementia.

[9] Takahashi T (2016). A potential contribution of antimicrobial peptide LL‐37 to tissue fibrosis and vasculopathy in systemic sclerosis. British Journal of Dermatology.

[10] Kahlenberg JM, Kaplan MJ (2013). Little peptide, big effects: the role of LL-37 in inflammation and autoimmune disease. The Journal of Immunology.

[11] Takahashi T, Gallo RL (2017). The Critical and Multifunctional Roles of Antimicrobial Peptides in Dermatology. Dermatol Clin.

[12] De Y (2000). LL-37, the neutrophil granule- and epithelial cell-derived cathelicidin, utilizes formyl peptide receptor-like 1 (FPRL1) as a receptor to chemoattract human peripheral blood neutrophils, monocytes, and T cells. J Exp Med.

[13] Lande R (2014). The antimicrobial peptide LL37 is a T-cell autoantigen in psoriasis. Nature communications.

[14] Lande R (2007). Plasmacytoid dendritic cells sense self-DNA coupled with antimicrobial peptide. Nature.

[15] Demaria O (2015). STING activation of tumor endothelial cells initiates spontaneous and therapeutic antitumor immunity. Proceedings of the National Academy of Sciences.

[16] Zhang L-j (2016). Antimicrobial Peptide LL37 and MAVS Signaling Drive Interferon-β Production by Epidermal Keratinocytes during Skin Injury. Immunity.

[17] Zhang X (2010). Dual functions of the human antimicrobial peptide LL-37—target membrane perturbation and host cell cargo delivery. Biochimica et Biophysica Acta (BBA)-Biomembranes.

[18] Bernard JJ (2012). Ultraviolet radiation damages self noncoding RNA and is detected by TLR3. Nature Medicine.

[19] Bals R, Wilson JM (2003). Cathelicidins-a family of multifunctional antimicrobial peptides. Cellular and Molecular Life Sciences.

[20] Roers A, Hiller B, Hornung V (2016). Recognition of Endogenous Nucleic Acids by the Innate Immune System. Immunity.

[21] Savarese E (2006). U1 small nuclear ribonucleoprotein immune complexes induce type I interferon in plasmacytoid dendritic cells through TLR7. Blood.

[22] Wang G (2008). Structures of human host defense cathelicidin LL-37 and its smallest antimicrobial peptide KR-12 in lipid micelles. J Biol Chem.

[23] Schmidt NW (2011). Criterion for amino acid composition of defensins and antimicrobial peptides based on geometry of membrane destabilization. Journal of the American Chemical Society.

[24] Schmidt NW, Wong GCL (2013). Antimicrobial peptides and induced membrane curvature: geometry, coordination chemistry, and molecular engineering. Current Opinion in Solid State and Materials Science.

[25] Lee, E. Y., Fulan, B. M., Wong, G. C. L. & Ferguson, A. L. Mapping membrane activity in undiscovered peptide sequence space using machine learning. *Proceedings of the National Academy of Sciences*, 201609893 (2016). 10.1073/pnas.1609893113 PMC5137689 27849600

[26] Brogden KA (2005). Antimicrobial peptides: pore formers or metabolic inhibitors in bacteria?. Nat Rev Microbiol.

[27] Di Nardo A (2007). Cathelicidin antimicrobial peptides block dendritic cell TLR4 activation and allergic contact sensitization. The Journal of Immunology.

[28] Gottschalk, S. & Thomsen, L. E. The Interaction of Antimicrobial Peptides with the Membrane and Intracellular Targets of Staphylococcus aureus Investigated by ATP Leakage, DNA-BindingAnalysis, and the Expression of a LexA-Controlled Gene, recA. *Antimicrobial Peptides: Methods and Protocols*, 297–305 (2017). 10.1007/978-1-4939-6737-7_21 28013513

[29] Schmidt NW (2015). Liquid-crystalline ordering of antimicrobial peptide-DNA complexes controls TLR9 activation. Nature materials.

[30] Lee EY (2016). A review of immune amplification via ligand clustering by self-assembled liquid–crystalline DNA complexes. Advances in colloid and interface science.

[31] Lee EY (2017). Crystallinity of Double-Stranded RNA-Antimicrobial Peptide Complexes Modulates Toll-Like Receptor 3-Mediated Inflammation. ACS nano.

[32] Limmon GV (2008). Scavenger receptor class-A is a novel cell surface receptor for double-stranded RNA. The FASEB Journal.

[33] Canton J, Neculai D, Grinstein S (2013). Scavenger receptors in homeostasis and immunity. Nat Rev Immunol.

[34] Ulvila J (2006). Double-stranded RNA is internalized by scavenger receptor-mediated endocytosis in Drosophila S2 cells. Journal of Biological Chemistry.

[35] MacLeod, D. T., Nakatsuji, T., Yamasaki, K., Kobzik, L. & Gallo, R. L. HSV-1 exploits the innate immune scavenger receptor MARCO to enhance epithelial adsorption and infection. *Nature communications* **4** (2013). 10.1038/ncomms2963 PMC3681428 23739639

[36] Tsuruoka H (2002). Scavenger receptor class B type I is expressed in cultured keratinocytes and epidermis. Regulation in response to changes in cholesterol homeostasis and barrier requirements. J Biol Chem.

[37] Peiser L, Mukhopadhyay S, Gordon S (2002). Scavenger receptors in innate immunity. Curr Opin Immunol.

[38] Ivanov, A. I. Pharmacological inhibition of endocytic pathways: is it specific enough to be useful? *Exocytosis and Endocytosis*, 15–33 (2008). 10.1007/978-1-59745-178-9_2 18369934

[39] Harper CB, Popoff MR, McCluskey A, Robinson PJ, Meunier FA (2013). Targeting membrane trafficking in infection prophylaxis: dynamin inhibitors. Trends Cell Biol.

[40] Gallo RL, Hooper LV (2012). Epithelial antimicrobial defence of the skin and intestine. Nat Rev Immunol.

[41] Yamasaki K (2007). Increased serine protease activity and cathelicidin promotes skin inflammation in rosacea. Nat Med.

[42] Lande R, Gilliet M (2010). Plasmacytoid dendritic cells: key players in the initiation and regulation of immune responses. Annals of the New York Academy of Sciences.

[43] Tursi SA (2017). Bacterial amyloid curli acts as a carrier for DNA to elicit an autoimmune response via TLR2 and TLR9. PLoS pathogens.

[44] Zanetti M, Gennaro R, Romeo D (1995). Cathelicidins: a novel protein family with a common proregion and a variable C‐terminal antimicrobial domain. FEBS Letters.

[45] Zaiou M, Gallo RL (2002). Cathelicidins, essential gene-encoded mammalian antibiotics. J Mol Med (Berl).

[46] Braff MH (2005). Structure-function relationships among human cathelicidin peptides: dissociation of antimicrobial properties from host immunostimulatory activities. J Immunol.

[47] Amarante MK, Watanabe MAE (2010). Toll-like receptor 3: involvement with exogenous and endogenous RNA. International Reviews of Immunology.

[48] Belgnaoui SM, Paz S, Hiscott J (2011). Orchestrating the interferon antiviral response through the mitochondrial antiviral signaling (MAVS) adapter. Current Opinion in Immunology.

[49] Saleh M-C (2006). The endocytic pathway mediates cell entry of dsRNA to induce RNAi silencing. Nature cell biology.

[50] Desmet CJ, Ishii KJ (2012). Nucleic acid sensing at the interface between innate and adaptive immunity in vaccination. Nature reviews. Immunology.

[51] Daaka Y (1998). Essential role for G protein-coupled receptor endocytosis in the activation of mitogen-activated protein kinase. Journal of Biological Chemistry.

[52] Ernst S, Zobiack N, Boecker K, Gerke V, Rescher U (2004). Agonist-induced trafficking of the low-affinity formyl peptide receptor FPRL1. Cellular and Molecular Life Sciences.

[53] Singh D, Qi R, Jordan JL, San Mateo L, Kao CC (2013). The human antimicrobial peptide LL-37, but not the mouse ortholog, mCRAMP, can stimulate signaling by poly(I:C) through a FPRL1-dependent pathway. J Biol Chem.

[54] Borkowski AW (2015). Toll-like receptor 3 activation is required for normal skin barrier repair following UV damage. Journal of Investigative Dermatology.

[55] Zhang L-j (2012). Coordinated regulation of transcription factor Bcl11b activity in thymocytes by the mitogen-activated protein kinase (MAPK) pathways and protein sumoylation. Journal of Biological Chemistry.

[56] Ilavsky J (2012). Nika: software for two-dimensional data reduction. Journal of Applied Crystallography.

[57] Hammersley AP (1997). FIT2D: an introduction and overview. European Synchrotron Radiation Facility Internal Report ESRF97HA02T.

[58] DeRouchey J, Netz RR, Rädler JO (2005). Structural investigations of DNA-polycation complexes. The European Physical Journal E: Soft Matter and Biological Physics.

[59] Warren BE (1941). X-ray diffraction in random layer lattices. Physical Review.

[60] Needleman DJ (2004). Higher-order assembly of microtubules by counterions: from hexagonal bundles to living necklaces. Proceedings of the National Academy of Sciences of the United States of America.

