# Supplementary material for: Cathelicidin promotes inflammation by enabling binding of self-RNA to cell surface scavenger receptors
Source: Sci Rep. 2018 Mar 5;8:4032. doi: 10.1038/s41598-018-22409-3 (PMC5838106; doi:10.1038/s41598-018-22409-3)
Supplement: Supplementary file 1 — supplementary information [file 41598_2018_22409_MOESM1_ESM.pdf]

Supplementary information

Cathelicidin promotes inflammation by enabling binding of self-RNA to cell surface scavenger receptors

Toshiya Takahashi[1], Nikhil Nitin Kulkarni[1], Ernest Y Lee[2], Ling-juan Zhang[1], Gerard C.L. Wong[2], Richard L. Gallo[1]*

1) Department of Dermatology, University of California, San Diego, La Jolla, CA 92037, United States, 2) Department of Bioengineering, University of California, Los Angeles, Los Angeles, CA 90095, United States

*Correspondence:

Dr. Richard L. Gallo,
Department of Dermatology, MC0869
University of California, San Diego
9500 Gilman Dr, La Jolla, CA 92093-0869
Telephone: 858- 822-4608
Fax: 858-822-6985
E-mail: rgallo@ucsd.edu

Supplementary Figures

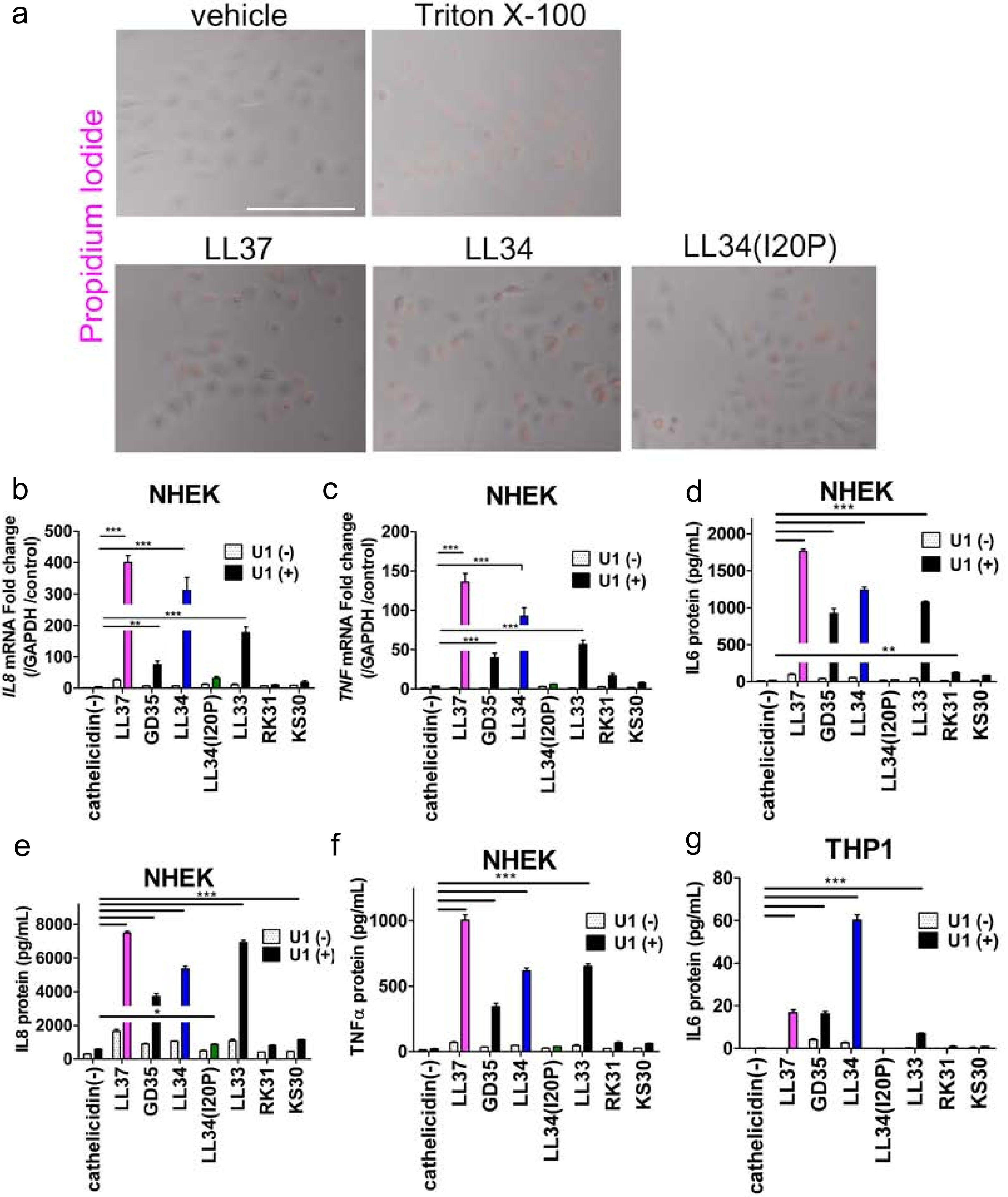

Figure S1. a) NHEKs were treated with cathelicidin peptides (2.5 μM) or Triton X-100 (0.1 %) for 15 minutes, and propidium iodide (0.5 μg/mL) immediately before observation. **b)** IL-8 mRNA abundance and **c)** tumor necrosis factor (TNF) α mRNA abundance in NHEKs after treatment with cathelicidin peptides (2.5 μM) for 10 minutes, then stimulated with U1 RNA (2.5 μg/mL) for a further 6 hours. (n = 3). **d)** IL-6 protein, **e)** IL-8 protein, and **f)** TNFα protein in the culture media of NHEKs treated as in **a** and **b**. **g)** IL-6 protein in the culture media of PMA-treated THP1 cells after treatment with cathelicidin peptides (3 μM) for 10 minutes, then stimulated with U1 RNA (12.5 μg/ml) overnight. (n = 3). Data presented are from one representative experiment of at least two independent experiments. Error bars are SEM of three biological replicates. *$P < 0.05$, **$P < 0.01$, ***$P < 0.001$ by two-way ANOVA with Bonferroni's post-hoc test.

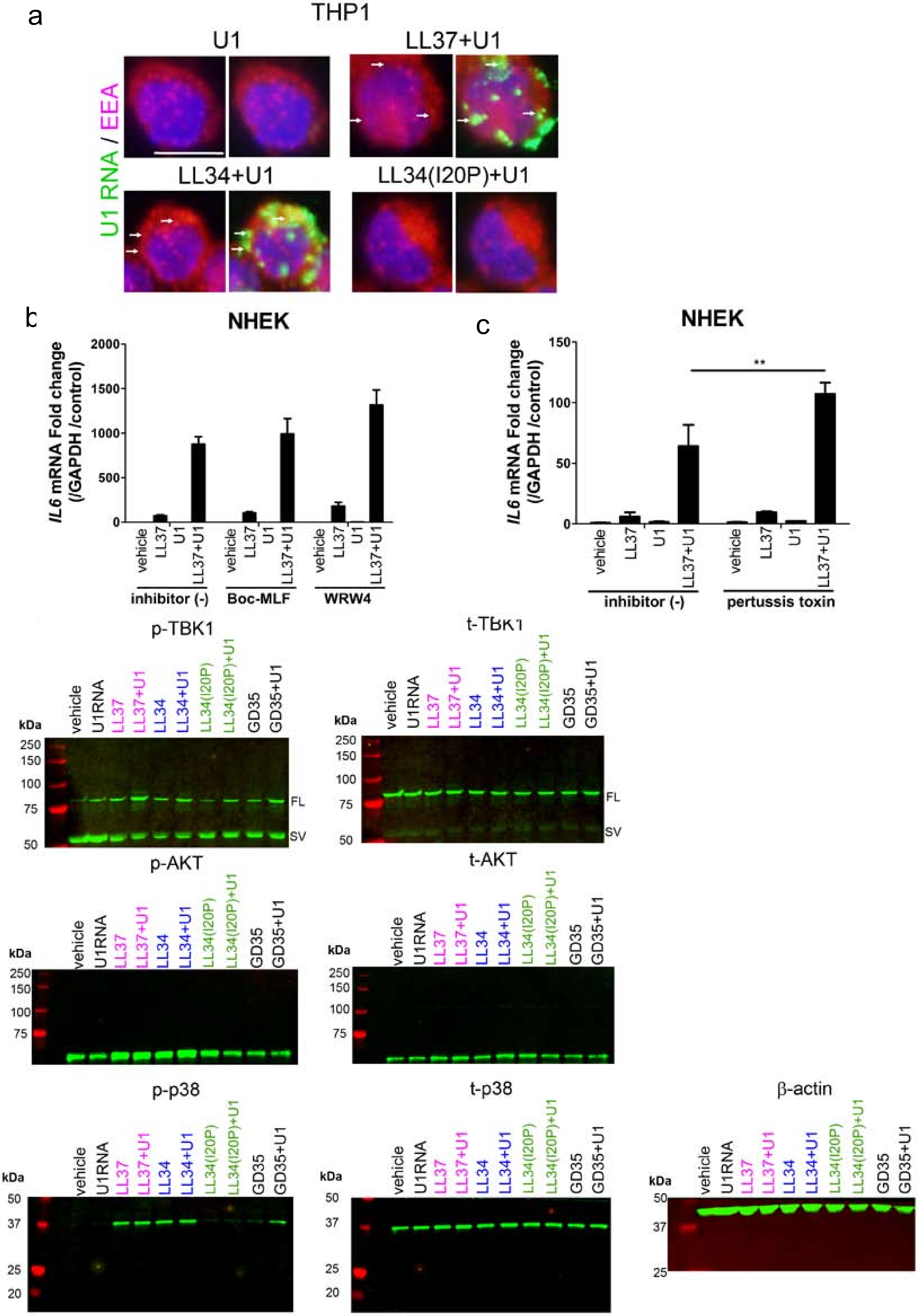
a
THP1
U1
LL37+U1
LL34+U1
LL34(I20P)+U1
U1 RNA / EEA
b
NHEK
IL6 mRNA Fold change (/GAPDH /control)
2000
1500
1000
500
0
vehicle
LL37
U1
LL37+U1
inhibitor (-)
Boc-MLF
WRW4
c
NHEK
IL6 mRNA Fold change (/GAPDH /control)
150
100
50
0
**
inhibitor (-)
pertussis toxin
p-TBK1
t-TBK1
kDa
250
150
100
75
50
FL
SV
vehicle
U1RNA
LL37
LL37+U1
LL34
LL34+U1
LL34(I20P)
LL34(I20P)+U1
GD35
GD35+U1
p-AKT
t-AKT
p-p38
t-p38
β-actin
37
25
20

Figure S2. a) PMA-treated THP1 cultured at 37 °C for 4 hours with U1 RNA and cathelicidin peptides. Early endosomal antigen 1 (EEA1) is stained red, U1 RNA is stained green and nuclei were visualized with DAPI (blue). Scale bar represents 10 μm. **b)** IL-6 mRNA in NHEKs cultured with FPR1 inhibitor Boc-MLF (1 μM) or FPR2 inhibitor WRW4 (1 μM) for 1 hour, treated with LL37 (2.5 μM) and U1 RNA (2.5 μg/mL) for a further 6 hours. (n = 3). **c)** IL-6 mRNA in NHEKs cultured with G-protein coupled receptor inhibitor pertussis toxin (0.5 μg/mL) for 1 hour, treated with LL37 (2.5 μM) and U1 RNA (2.5 μg/mL) for a further 6 hours. (n = 3). Error bars are SEM of biological replicates. **d)** NHEKs were pretreated with cathelicidin peptides (2.5μM) for 10 minutes, then stimulated with U1 RNA (2.5 μg/mL) for a further 30 minutes (for AKT and p38) or 2 hours (for TBK and β–actin). Cell extracts were subjected to immunoblotting analyses using indicated antibodies after cutting each membrane at 50kDa (30 minutes upper: AKT, 30 minutes below: p38, 2 hours upper: TBK1, and 2 hours below: β–actin). Cropped blots are presented in Figure 2g. Abbreviations: SV: spliced variant; FL: full length.

Data presented are from one representative experiment of at least two independent experiments. Error bars are SEM of three biological replicates.*P < 0.05, **P < 0.01, ***P < 0.001 by two-way ANOVA with Bonferroni's post-hoc test.

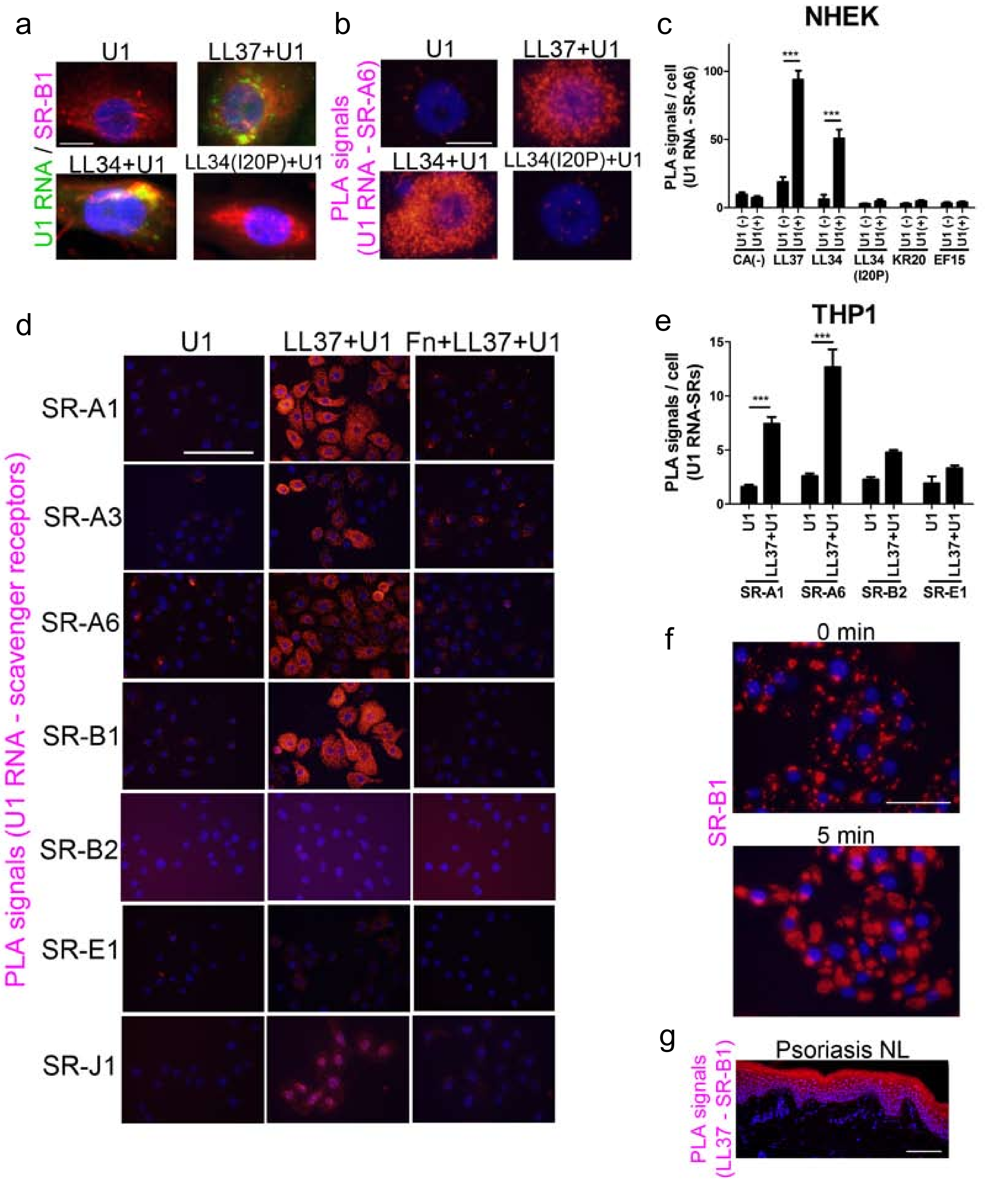
a
U1
LL37+U1
LL34+U1
LL34(I20P)+U1
U1 RNA / SR-B1
b
U1
LL37+U1
LL34+U1
LL34(I20P)+U1
PLA signals (U1 RNA - SR-A6)
c
NHEK
PLA signals / cell (U1 RNA - SR-A6)
***
***
U1 (-) U1 (+)
CA(-) LL37 LL34 LL34 (I20P) KR20 EF15
d
U1
LL37+U1
Fn+LL37+U1
SR-A1
SR-A3
SR-A6
SR-B1
SR-B2
SR-E1
SR-J1
PLA signals (U1 RNA - scavenger receptors)
e
THP1
PLA signals / cell (U1 RNA-SRs)
***
***
U1
LL37+U1
SR-A1 SR-A6 SR-B2 SR-E1
f
0 min
5 min
SR-B1
g
Psoriasis NL
PLA signals (LL37 - SR-B1)

Figure S3. a) NHEKs cultured at 4 °C for 10 minutes with U1 RNA and cathelicidin peptide. SR-B1 is stained red, U1 RNA is stained green and nuclei were visualized with DAPI (blue). Scale bar represents 10 μm. **b)** Proximity ligation assay for SR-A6 and U1 RNA. NHEKs were cultured with various cathelicidin peptides (2.5 μM) for 10 minutes, then with biotinylated U1 RNA (2.5 μg/mL) at 4 °C for 1 hour before physical proximity of SR-A6 and U1 RNA was determined using a fluorescence-based PLA that produces a red fluorescent signal. Nuclei (blue) are counterstained with DAPI. Scale bar represents 10 μm. **c)** Signal count of **b** in 5 visual fields. **d)** Proximity ligation assay as in **b**, but reaction is detecting proximity of U1 RNA and the indicated scavenger receptors. Scale bar represents 100 μm. **e)** Signal count of proximity ligation assay for U1 RNA and the indicated scavenger receptors. PMA-treated THP1 were cultured with various cathelicidin peptides (3 μM) for 10 minutes, then with biotinylated U1 RNA (2.5 μg/mL) at 4 °C for 1 hour before physical proximity of U1 RNA and scavenger receptors was determined using a fluorescence-based PLA, each count is PLA complexes observed in 5 visual fields. **f)** NHEKs cultured at 37 °C that were stained immediately after or after 5 minutes with U1 RNA and LL37, demonstrating enlarged complex sizes of SR-B1 after 5 minutes at similar focal planes. SR-B1 is stained red and nuclei were visualized with DAPI (blue). Scale bar represents 100 μm. **g)** Skin sections from non-lesional skin of

patients with psoriasis was fixed and PLA performed for LL37 and SR-B1. Red fluorescent signal defines cells where LL37 and SR-B1 are within <40 nm of each other. Nuclei (blue) are counterstained with DAPI. Scale bars represent 100 μm.

Data presented are from one representative experiment of at least two independent experiments. Error bars are SEM of three biological replicates. *P < 0.05, **P < 0.01, ***P < 0.001 by two-way ANOVA with Bonferroni's post-hoc test.

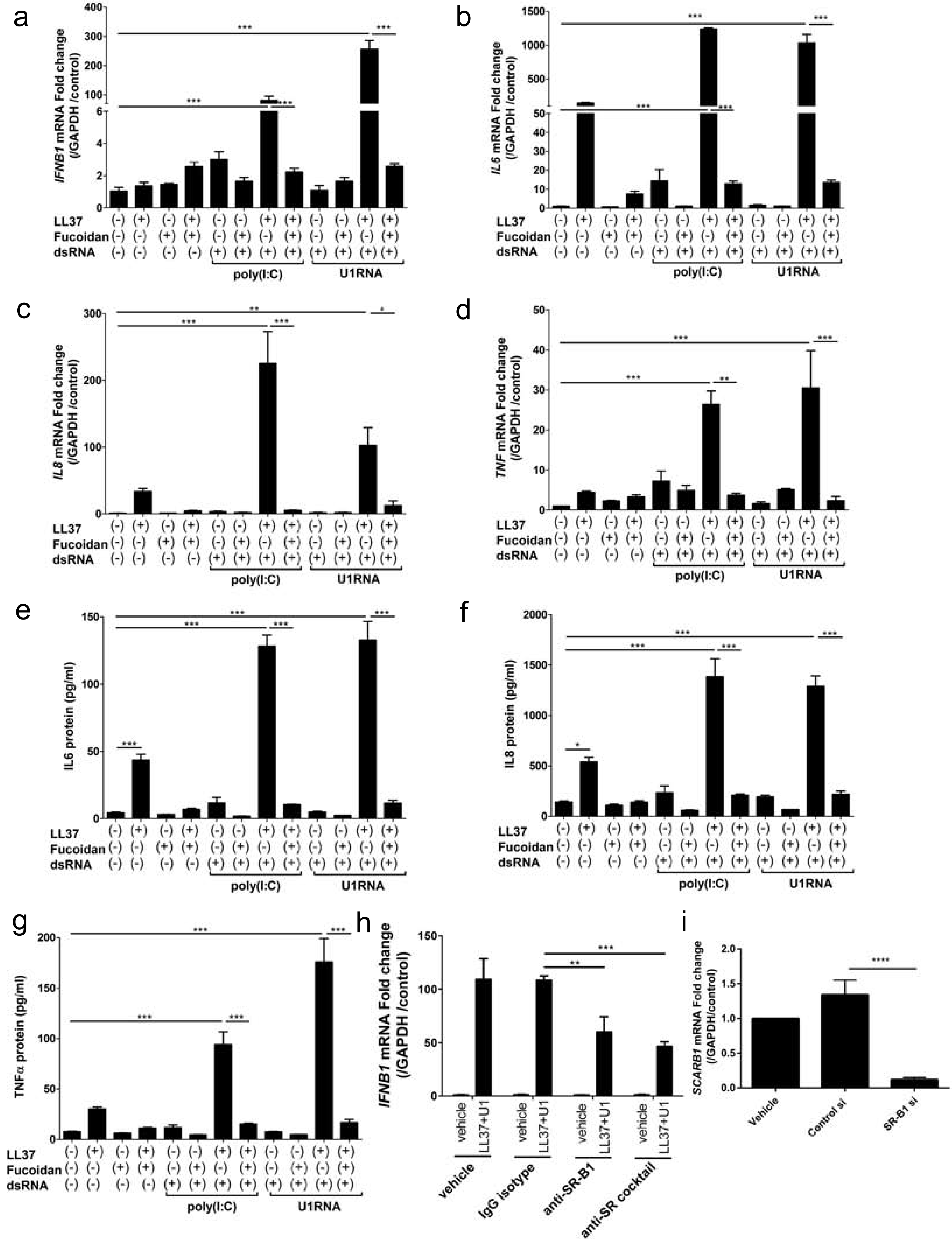

a
IFNB1 mRNA Fold change (/GAPDH /control)
b
IL6 mRNA Fold change (/GAPDH /control)
c
IL8 mRNA Fold change (/GAPDH /control)
d
TNF mRNA Fold change (/GAPDH /control)
e
IL6 protein (pg/ml)
f
IL8 protein (pg/ml)
g
TNFα protein (pg/ml)
h
IFNB1 mRNA Fold change (/GAPDH /control)
i
SCARB1 mRNA Fold change (/GAPDH/control)
LL37
Fucoidan
dsRNA
poly(I:C)
U1RNA
vehicle
LL37+U1
IgG isotype
anti-SR-B1
anti-SR cocktail
Vehicle
Control si
SR-B1 si

Figure S4. a) IFN-β1 mRNA, **b)** IL-6 mRNA, **c)** IL-8 mRNA and **d)** TNFα mRNA in NHEKs cultured with fucoidan (10 μg/ml) for 10 minutes, treated with LL37 (2.5 μM) and poly(I:C) (0.3 μg/mL) or U1 RNA (2.5 μg/mL) for a further 6 hours. (n = 3). **e)** IL-6 protein, **f)** IL-8 protein, and **g)** TNFα protein in the culture media of NHEKs treated as in **a-d**. **h)** IFN-β1 mRNA in NHEKs cultured in vehicle alone or with antibodies for SR-B1, a mixture of antibodies for 7 scavenger receptors, SR-A1, A3, A6, B1, B2, E1, and J1 (SR cocktail), or IgG isotype (control) at 12 μg/mL for 5 minutes following cycloheximide (10 μg/mL) for 30 minutes, then treated with LL37 (2.5 μM) for 10 minutes, and U1 RNA (2.5 μg/mL) for a further 6 hours. **i)** SCARB1 mRNA in NHEKs following siRNA knockdown of gene coding SR-B1 (SCARB1). (n = 3). Data presented are from one representative experiment of at least two independent experiments. Error bars are SEM of three biological replicates. *$P < 0.05$, **$P < 0.01$, ***$P < 0.001$ by two-way ANOVA with Bonferroni's post-hoc test.

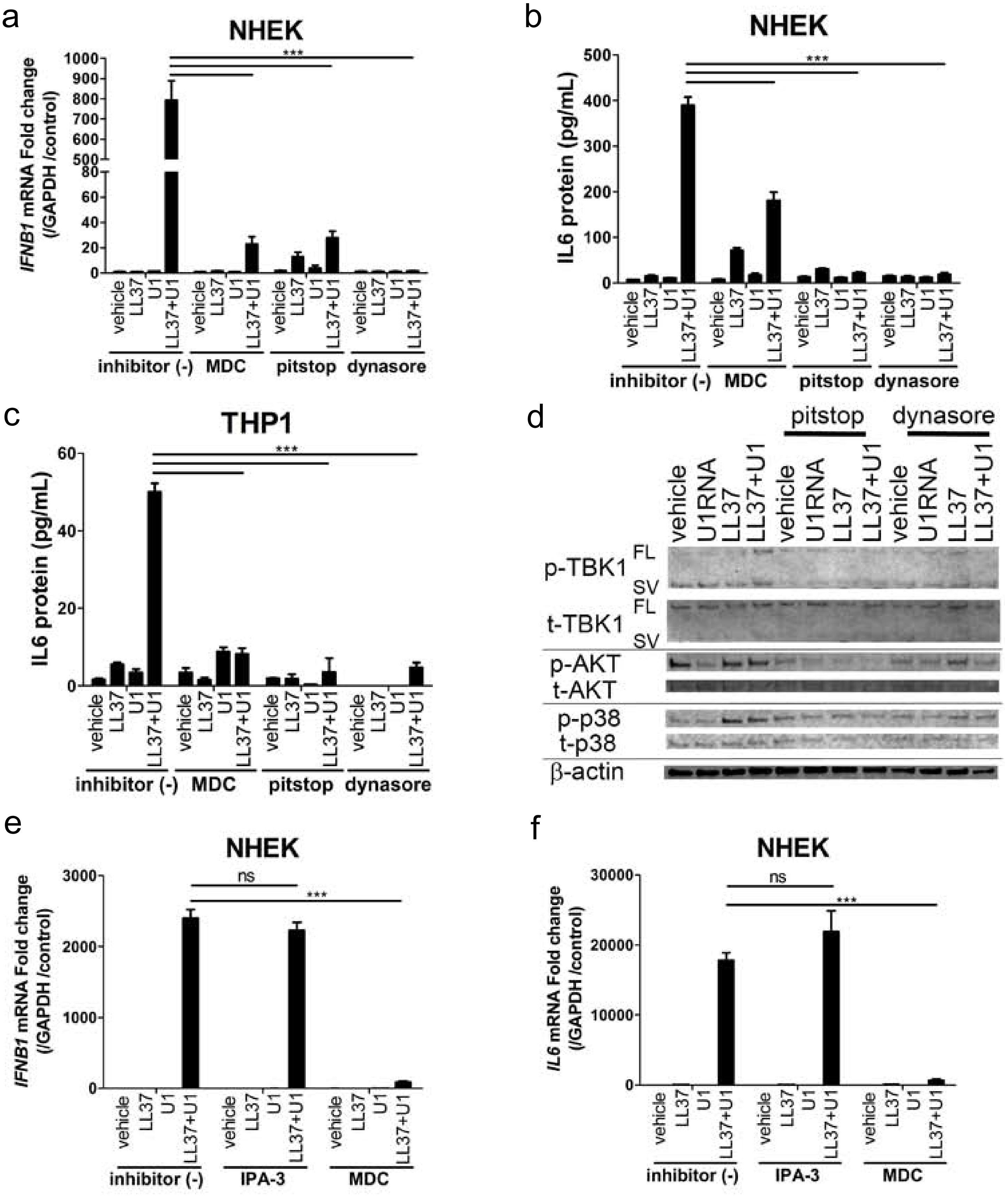
a
NHEK
IFNB1 mRNA Fold change (/GAPDH /control)
***
vehicle
LL37
U1
LL37+U1
inhibitor (-)
MDC
pitstop
dynasore
b
NHEK
IL6 protein (pg/mL)
***
c
THP1
IL6 protein (pg/mL)
***
d
pitstop
dynasore
vehicle
U1RNA
LL37
LL37+U1
p-TBK1
t-TBK1
FL
SV
p-AKT
t-AKT
p-p38
t-p38
β-actin
e
NHEK
IFNB1 mRNA Fold change (/GAPDH /control)
ns
***
IPA-3
f
NHEK
IL6 mRNA Fold change (/GAPDH /control)
ns
***

Figure S5. a) IFN-β1 mRNA in NHEKs cultured with various endocytosis inhibitors (Monodansylcadaverine (MDC): 200 μM, Pitstop-2^{TM} 25 μM, dynasore: 80 μM) for 30 minutes, treated with LL37 (2.5 μM) and U1 RNA (2.5 μg/mL) for a further 6 hours. (n = 3). **b)** IL-6 protein in the culture media of NHEKs treated as in **a**. **c)** IL-6 protein in the culture media of PMA-treated THP1 cells after treatment with various endocytosis inhibitors (Monodansylcadaverine (MDC): 200 μM, Pitstop-2^{TM} 25 μM, dynasore: 80 μM) for 30 minutes, treated with LL37 (3 μM) and U1 RNA (12.5 μg/mL) overnight. (n = 3). **d)** NHEKs were exposed to endocytosis inhibitors (Pitstop-2^{TM} 25 μM, dynasore: 80 μM) for 30 minutes, and treated with LL37 (2.5μM) for 10 minutes, and stimulated with U1 RNA (2.5 μg/mL) for a further 30 minutes (for AKT and p38) or 2 hours (for TBK and βactin). Cell extracts were subjected to immunoblotting analyses using indicated antibodies. Abbreviations: SV: spliced variant; FL: full length. **e)** IFN-β1 mRNA and **f)** IL-6 mRNA in NHEKs cultured with macropinocytosis inhibitor IPA-3 (2.8 nM) or MDC (200 μM) for 30 minutes, treated with LL37 (2.5 μM) and U1 RNA (2.5 μg/mL) for a further 6 hours. (n = 3). Data presented are from one representative experiment of at least two independent experiments. Error bars are SEM of three biological replicates. *P < 0.05, **P < 0.01, ***P < 0.001 by two-way ANOVA with Bonferroni's post-hoc test.

Supplementary Video

Confocal images of NHEKs cultured at 37 °C that were stained **a)** immediately after and **b)** after 5 minutes with U1 RNA and LL37. SR-B1 is stained red and nuclei were visualized with DAPI (blue). Scale bar represents 10 μm.

Supplementary Tables

Table S1. List of antibodies.

**Antibodies**	**Source**	**Identifier**	**dilution**
AKT	Cell Signaling	Cat#2920; RRID:AB_1147620	1:1000
β-actin	Santa Cruz	Cat#sc-47778; RRID: AB_626632	1:2000
biotin	Santa Cruz	Cat#sc-101339; RRID:AB_1119609	1:200
CD36 (SR-B2)	Abcam	Cat#ab133625;	1:200
EEA1	Cell Signaling	Cat#3288; RRID:AB_2096811	1:200
LL37	Santa Cruz	Cat#sc-166770; RRID:AB_2068692	1:200
LOX1 (SR-E1)	Santa Cruz	Cat#sc-20753; RRID:AB_2236501	1:100
MARCO (SR-A6)	Santa Cruz	Cat#sc-68913; RRID:AB_2140586	1:200
MSR1 (SR-A1)	Abcam	Cat#ab123946; RRID:AB_10974791	1:200
p38 MAPK	Cell Signaling	Cat#9212; RRID:AB_330713	1:500
Phospho-AKT	Cell Signaling	Cat#4060; RRID:AB_2315049	1:2000
Phospho-p38 MAPK	Cell Signaling	Cat#9216; RRID:AB_33129	1:1000
Phospho-TBK1	Cell Signaling	Cat#5483; RRID:AB_10693472	1:1000
RAGE (SR-J1)	Abcam	Cat#ab3611; RRID:AB_303947	1:200
SCARA3 (SR-A3)	Sigma	Cat#SAB2700220;	1:200
SCARB1 (SR-B1)	Novus Biologicals	Cat#NB400-104; RRID:AB_2182824	1:200

TBK1	Cell Signaling	Cat#3504; RRID:AB_2255663	1:1000
anti-Mouse IgG, Alexa Fluor™ 488	Thermofisher scientific	Cat#A21202;	1:500
anti-Rabbit IgG, Alexa Fluor™ 568	Thermofisher scientific	Cat#A10042;	1:500
IRDye® 800CW anti-Mouse IgG	LI-COR	Cat#926-32210;	1:3000
IRDye® 800CW anti-Rabbit IgG	LI-COR	Cat#926-32213;	1:3000
Duolink® In Situ PLA® Probe Anti-Mouse MINUS	Sigma	Cat#DUO92004	N/A
Duolink® In Situ PLA® Probe Anti-Rabbit PLUS	Sigma	Cat#DUO92002	N/A

Table S2. Assay ID of quantitative PCR primers and probes.

Gene	Refseq	Taqman® Gene Expression Assay ID
GAPDH	NM_001256799.2 NM_001289745.1 NM_001289746.1 NM_002046.5	Hs02758991_g1
IFNB1	NM_002176.3	Hs01077958_s1
IL6	NM_000600.4	Hs00985639_m1
IL8	NM_000584.3	Hs00174103_m1
SRA6	NM_006770.3 XM_011512082.1 XM_011512083.2 XM_017005171.1	Hs00198937_m1
SRB1	NM_001082959.1 NM_005505.4	Hs00969821_m1
TNF	NM_000594.3	Hs01113624_g1
**Gene**	**Refseq**	**PrimeTime® qPCR Assay ID**
DNM1	NM_004408 NM_001005336	Hs. PT. 58. 25262501
CLTC	NM_004859	Hs. PT. 58. 280806
